# Hydrophilic Copolymers with Hydroxamic Acid Groups as a Protective Biocompatible Coating of Maghemite Nanoparticles: Synthesis, Physico-Chemical Characterization and MRI Biodistribution Study

**DOI:** 10.3390/pharmaceutics15071982

**Published:** 2023-07-19

**Authors:** Hana Charvátová, Zdeněk Plichta, Jiřina Hromádková, Vít Herynek, Michal Babič

**Affiliations:** 1 Institute of Macromolecular Chemistry, Czech Academy of Sciences, Heyrovského Náměstí 2, 162 06 Prague, Czech Republic; charvatova@imc.cas.cz (H.C.);; 2 Center for Advanced Preclinical Imaging (CAPI), First Faculty of Medicine, Charles University, Salmovská 3, 120 00 Prague, Czech Republic; vit.herynek@lf1.cuni.cz

**Keywords:** superparamagnetic iron oxide nanoparticles, maghemite, hydroxamic acid, non-fouling surface, polymer coating, MRI, contrast agents

## Abstract

Superparamagnetic iron oxide nanoparticles (SPION) with a “non-fouling” surface represent a versatile group of biocompatible nanomaterials valuable for medical diagnostics, including oncology. In our study we present a synthesis of novel maghemite (γ-Fe_2_O_3_) nanoparticles with positive and negative overall surface charge and their coating by copolymer P(HPMA-*co*-HAO) prepared by RAFT (reversible addition–fragmentation chain-transfer) copolymerization of N-(2-hydroxypropyl)methacrylamide (HPMA) with N-[2-(hydroxyamino)-2-oxo-ethyl]-2-methyl-prop-2-enamide (HAO). Coating was realized via hydroxamic acid groups of the HAO comonomer units with a strong affinity to maghemite. Dynamic light scattering (DLS) demonstrated high colloidal stability of the coated particles in a wide pH range, high ionic strength, and the presence of phosphate buffer (PBS) and serum albumin (BSE). Transmission electron microscopy (TEM) images show a narrow size distribution and spheroid shape. Alternative coatings were prepared by copolymerization of HPMA with methyl 2-(2-methylprop-2-enoylamino)acetate (MMA) and further post-polymerization modification with hydroxamic acid groups, carboxylic acid and primary-amino functionalities. Nevertheless, their colloidal stability was worse in comparison with P(HPMA-*co*-HAO). Additionally, P(HPMA-*co*-HAO)-coated nanoparticles were subjected to a bio-distribution study in mice. They were cleared from the blood stream by the liver relatively slowly, and their half-life in the liver depended on their charge; nevertheless, both cationic and anionic particles revealed a much shorter metabolic clearance rate than that of commercially available ferucarbotran.

## 1. Introduction

Superparamagnetic iron oxide nanoparticles (SPION), mainly magnetite (Fe_3_O_4_) and maghemite (γ-Fe_2_O_3_), have been extensively studied for possible applications in biotechnology and biosciences. Since these nanoparticles exhibit high magnetization, large specific surface [1] and low renal accumulation (determined by coating) [2], their uses mainly include magnetic separations [3,4,5], sensors [6,7,8], induced hyperthermia cancer treatments [9,10,11], targeted drug and gene delivery [12,13,14,15] and as contrast agents for magnetic resonance imaging (MRI) [16,17,18], as well as magnetic particle imaging (MPI) [16,17,18,19,20,21].

Magnetic resonance imaging (MRI) is one of the most frequently used radiological methods for in vivo imaging of physiological, pathological and oncological structures. MRI is an imaging method based on the interaction of an external magnetic field with nuclei having non-zero spin (e.g., hydrogen ^1^H, fluorine ^19^F, phosphorus ^31^P). MRI minimizes side-effects for a patient due to the absence of ionizing radiation; a radio frequency (RF) pulse employed in the presence of the magnetic field allows the obtaining of anatomical as well as functional information and produces cross-sectional images of the body in any plane. Signal intensity depends mainly on the concentration of detected nuclei within the particular tissue and its magnetic properties. For this reason, one of the most frequently investigated nuclei in clinical practice is hydrogen ^1^H. Its high concentration of which in living organisms (present in molecules of water, fats and in many biomolecules), together with the favorable internal physical properties (*s* = 1/2, *γ_n_*/2π = 42.58 MHz/T) of the nucleus, allows its easy detection at relatively low magnetic fields. Valuable anatomical and functional information can be obtained by measuring the mobility of the water molecules due to their diffusion or flow, and by measuring the relaxation times of the ^1^H nuclei [22]. To improve contrast, and therefore image quality, the use of contrast agents (CA) has proven to be advantageous. Gadolinium chelates are nowadays the most commonly used ones in everyday clinical practice. Interactions of a gadolinium complex with water molecules shortens the relaxation times of proton nuclei in body tissues, which leads to signal enhancement for T_1_-weighted images. Examples of commercially available gadolinium macrocycles are gadobutrol (Gadovist^®^), gadoteridol (Prohance^®^) and gadoterate meglumine (Dotarem^®^), while from “linear” complexes there can be mentioned gadobenic acid (Multihance^®^), gadodiamide (Omniscan^®^), gadopentetate dimeglumine (Magnevist^®^), gadoversetamide (Optimark^®^) and gadoxetic acid (Primovist^®^) [23]. Nevertheless, most of the gadolinium chelates cannot be used directly for a preferential imaging of specific areas or compartments, and can be partially toxic due to a possible release of gadolinium ions. In the literature, researchers report gadolinium deposits measured in brain and bones, denoted as “gadolinium deposition disease”, in patients with normal renal function [24,25]. Patients with chronic renal disfunction have an increased probability of gadolinium-induced nephrotoxicity development [26]. This is the reason why superparamagnetic iron oxide nanoparticles, which do not contain any toxic metal, are intensively studied in order to develop contrast agents with better biocompatibility and specificity. In comparison with gadolinium chelates, which are low-molecular-weight compounds, coated superparamagnetic iron oxide nanoparticles have much larger diameter and thus, after intravenous or intraarterial administration, can passively accumulate in solid tumors due to the enhanced permeability and retention effect (EPR). Tumors often display specifically increased vascularization, which is accompanied by increased blood flow. Increased vascular permeability and poor lymphatic drainage enable enhanced capture of the larger particles into tumors [22].

There is a plethora of synthetic pathways leading to iron oxide nanoparticles, including the sol–gel method [27], hydrothermal method [28], flow injection method [29], electrochemical method [30,31], aerosol/vapor-phase method [32], sonochemical decomposition method [33,34], supercritical fluid method [35], synthesis using nanoreactors method [36] and microbial method [37,38]. However, thermal decomposition of iron-organic precursors [39,40] and coprecipitation of ferrous and ferric salts with an aqueous base [41,42] became the most-preferred ways.

Coprecipitation of iron salts, FeCl_2_ and FeCl_3_, is a simple, easily scalable preparation method, and the resulting magnetic nanoparticles exhibit narrow size distribution and high magnetization values. When the solutions of iron salts are separately treated with a base, it leads to the formation of iron hydroxide intermediate species. After their mixing, these intermediates consequently form magnetite nanoparticles with more precise composition and structure [43]. The chemical stability of the product can be enhanced by oxidation to maghemite, which is considered to be a more stable and more biocompatible alternative to the magnetite, which can provoke oxidation of some biomolecules [3,44].

The overall charge of the nanoparticles has a great impact on their behavior in the body. For instance, endocytosis into cell endosomes can be facilitated by several transfection agents [45], which are mostly cationic [46]. Complexing of the above-mentioned polymers, containing partially positively charged groups, with the mainly anionic surface of nanoparticles takes place due to multiple electrostatic interactions. Nevertheless, this type of bond, usually stable in salt-free conditions, does not necessarily persist in the presence of electrolytes [47].

Iron oxide nanoparticles in their crude and uncoated form are rather unstable and tend to aggregate, especially in electrolytes and biological fluids. Uncoated particles also show rather high cytotoxicity due to protein [48] and DNA [2,49] damage and change of mitochondrial membrane potential [29]. A polymer coating can reduce toxicity and improve colloidal and physical stability, as well as the water-dispersibility of the nanoparticles. Simultaneously, the coating can prolong their half-life in biological systems and enable further modification of their surface via the functional groups present in a biocompatible polymer chain [50,51,52]. Several materials for the surface coating of magnetic particles have been reported in the literature, e.g., dextran [53,54], chitosan [55,56], polyethylene glycol [57,58], poly(L-lysine) [41,59], poly(*N,N*-dimethylacrylamide) [47] and P(HPMA) [60].

Generally, a chelating ligand forming a five-membered ring with the surface iron atom seems to be ideal for obtaining tailored functional groups with a strong affinity to the iron oxide nanoparticle’s surface. Outstanding metal-complexing ability, especially for Fe(III), is exhibited by hydroxamic acids, which are widely used as anti-corrosives and pharmaceuticals [61]. Compounds bearing the hydroxamic acid group play a crucial role in natural iron metabolism [62] and show a high capability to scavenge iron from the bloodstream in cases of an overloading induced by either a disease or iron poisoning [63]. This functional group can be also found in siderophores synthesized by microorganisms to increase iron bioavailability [64,65].

**Figure 1:**
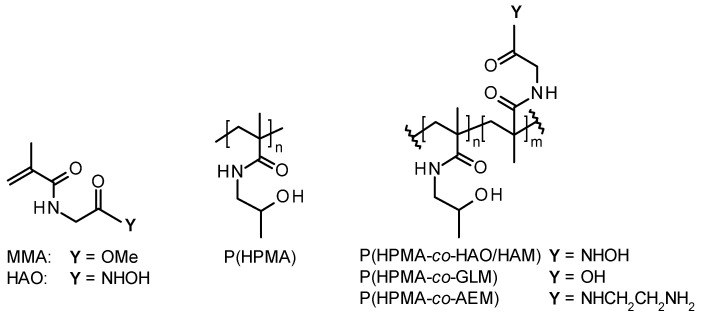
Monomers, P(HPMA) and novel copolymers as ligands for iron oxide nanoparticles.

In our previous study [66], we presented promising physico-chemical data (magnetometry, Mössbauer spectroscopy and MPI) of maghemite nanoparticles coated by the copolymer containing a hydroxamic acid unit. The data documented no adverse effects of the coating on magnetic properties or the particles’ stability under strong applied fields of MPI. Encouraged by these results, we decided to extend our research of the coated nanoparticles. Thus, we synthesized and characterized maghemite nanoparticles with positive as well as negative total surface charge and documented their colloidal behavior. The polymer coating was synthesized by reversible addition–fragmentation chain transfer (RAFT) copolymerization of HPMA (*N*-(2-hydroxypropyl)methacrylamide) with *N*-[2-(hydroxyamino)-2-oxo-ethyl]-2-methyl-prop-2-enamide (HAO). Additionally, we copolymerized HPMA with MMA (methyl 2-(2-methylprop-2-enoylamino)acetate) and post-synthetically modified the copolymer by the introduction of amine as well as carboxylic and hydroxamic acid groups (Figure 1). The reason for introduction of amine, carboxylic acid and, mainly, hydroxamic acid functional groups to the polymer structure relates to an attempt to synthesize coated magnetic nanoparticles stable over a wide range of conditions. A slight disadvantage of the parent polymer is a lack of biodegradability; nevertheless, this can be easily overcome by the use of polymers with molecular weights below the limit of renal filtration (*M_w_* approx. 5 × 10^4^) [67].

As a proof of principle, the chosen coated nanoparticles were tested as contrast agents for MRI. Their elimination and persistence in tissues, as well as the rate of their elimination were compared with a commercially available contrast agent [68].

## 2. Materials and Methods

### 2.1. Chemicals

FeCl_3_·6H_2_O, FeCl_2_·4H_2_O, trisodium citrate dihydrate, glycine methyl ester hydrochloride, triethylamine, methacryloyl chloride, 1-phenyl-3-pyrazolidinone, NH_2_OH·HCl, 2,2′-azobis(2-methylpropionitrile) (AIBN), 4-Cyano-4-(phenylcarbonothioylthio)pentanoic acid (CTPA), phosphate buffered saline (PBS), 1,2-diaminoethane and silica gel (pore size 60 Å) were purchased from Sigma-Aldrich (St. Louis, MO, USA). Aqueous ammonia, hydrogen peroxide, HCl, 1,2-dichloroethane (DCE), NaCl, methanol, ethyl acetate, NaOH and diethyl ether were purchased from Lach-Ner, Neratovice, Czech Republic. Albumin was obtained from Serva (Heidelberg, Germany). Sephadex^®^ G-20 was purchased from GE Healthcare (Chicago, IL, USA). Aqueous solutions of FeCl_3_·6H_2_O and FeCl_2_·4H_2_O were purified by centrifugation for 10 min at 4400 rpm. 1,2-Dichlorethane, triethylamine, 2,2′-azobis(2-methylpropionitrile) (AIBN) and 4-Cyano-4-(phenylcarbonothioylthio)pentanoic acid (CTPA) were distilled before use. Ethanol was rectified. *N*-(2-hydroxypropyl)methacrylamide) (HPMA) was prepared according to a literature procedure [69]. All aqueous solutions in this study were prepared from ultrapure Q-water (18.2 MΩ) ultrafiltered on a Milli-Q Gradient A10 system (Milipore, Molsheim, France).

### 2.2. Synthesis of Maghemite Nanoparticles

The aqueous FeCl_3_·6H_2_O solution (0.2 M, 100 mL) was treated with aqueous ammonia (0.5 M, 100 mL) under sonication for 2 min, and an FeCl_2_·4H_2_O solution (0.2 M, 55 mL) was added. The resulting brownish dispersion was poured into the excess of the aqueous ammonia (0.5 M, 250 mL) under stirring, which led to the formation of black magnetite particles. The mixture was stirred for 1 h and left to sediment. The supernatant was decanted, and the particles were purified by stirring with water followed by repeated (5×) magnetic separation and decantation. A solution of H_2_O_2_ (3%, 6 mL) was added to the colloid under sonication within 10 min, which was accompanied by vigorous gas evolution. The colloid was left to separate on a magnetic separator for four days; brown supernatant was sucked up, and the nanoparticles were dispersed in water (20 mL). A cationic colloid (denoted as γ-Fe_2_O_3_^⨁^) was obtained by the addition of HCl (0.2 M, 9 mL) under sonication for 2 min. In the synthesis of anionic colloid (denoted as γ-Fe_2_O_3_^⊖^), hydrogen peroxide (3%, 6 mL) was added after stabilization of the particles by the trisodium citrate (0.1 M, 12 mL) solution. The resulting colloid was purified as described above. Both colloids were filtered through a 0.45 μm syringe PVDF filter and the concentration was set to 60 mg/mL. For characterization data, see Table 1.

### 2.3. Synthesis of Methyl 2-(2-Methylprop-2-enoylamino)acetate (MMA)

**Figure 2:**
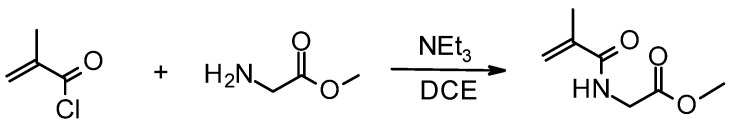
Synthesis of methyl 2-(2-methylprop-2-enoylamino)acetate) (MMA).

A two-necked 250 mL flask equipped with a stirring bar was loaded with glycine methyl ester hydrochloride (1.26 g; 10 mmol), dichloroethane (100 mL) and triethylamine (3.1 mL; 22 mmol) under an argon atmosphere. Methacryloyl chloride (1.1 mL; 11 mmol) was added dropwise, and the color changed to light orange (Figure 2). After 2 h, the white solid was separated, the orange solution was extracted twice with saturated NaCl solution, and the resulting crude MMA was dried for 16 h with solid NaCl. The solvent was vacuum-evaporated, and the residual orange oil was purified by chromatography on a silica gel column using dichloroethane/methanol 20/1 (*v*/*v*) mixture as the eluent.

Yield: 0.94 g (65%). ^1^H NMR (CDCl_3_): *δ* 1.95 (s, 3 H, CH_3_), 3.77 (s, 3 H, OCH_3_), 4.10 (d, ^3^*J*_HH_ = 5.1 Hz, 2 H, CH_2_), 5.57 (d, ^2^*J*_HH_ = 112 Hz, 2 H, CH_2_=C), and 6.35 (br s, 1 H, NH).

### 2.4. Synthesis of N-[2-(Hydroxyamino)-2-oxo-ethyl]-2-methyl-prop-2-enamide (HAO)

**Figure 3:**
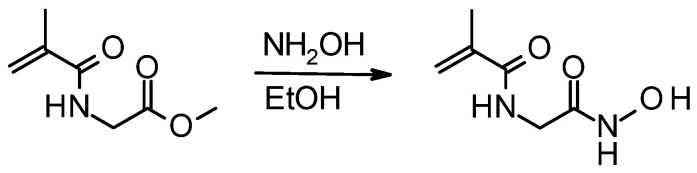
Synthesis of *N*-[2-(hydroxyamino)-2-oxo-ethyl]-2-methyl-prop-2-enamide (HAO).

A flask equipped with a magnetic stirrer was loaded with MMA (0.508 g, 3.5 mmol), and 1-phenyl-3-pyrazolidinone (20 mg) as a polymerization inhibitor. Afterwards, ethanol (20 mL) was introduced, followed by the aqueous solution of hydroxylamine (4.4 mL, 2.4 M) (Figure 3). A color change was observed after the addition of hydroxylamine—the initially yellowish solution changed its color to orange. The flask was flushed with argon and sealed. The reaction mixture was stirred at room temperature for 16 h in the dark. The crude product was pre-adsorbed on silica gel and purified by column chromatography with dichloroethane/methanol 5/1 as the eluent.

Yield: 0.290 g, 57%. ^1^H NMR (DMSO): *δ* 1.86 (s, 3 H, CH_3_), 3.63 (d, ^3^*J*_HH_ = 6.0 Hz, 2 H, CH_2_), 5.54 (d, ^2^*J*_HH_ = 112 Hz, 2 H, CH_2_=C), 8.13 (br s, 1 H, OH), 8.78 (br s, 1 H, NH), and 10.50 (br s, 1 H, NH).

### 2.5. RAFT Polymerization of HPMA and Its Copolymerization with MMA or HAO

**Figure 4:**
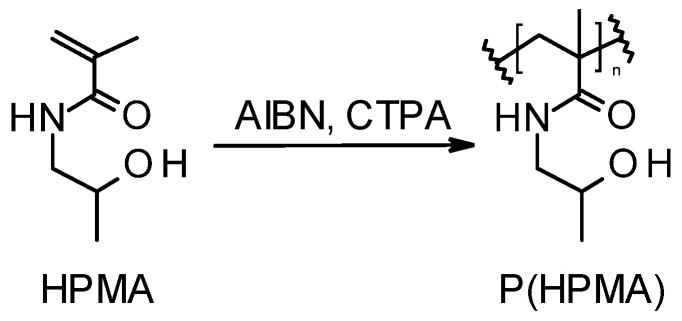
Polymerization of HPMA.

A dry 100 mL flask was loaded with a solution of HPMA (1 g, 7.0 mmol), AIBN (2.1 mg, 0.013 mmol) and CTPA (7.0 mg, 0.025 mmol) in ethanol (9 mL), and HCl (5 M, 50 μL) was added under argon atmosphere (Figure 4). The resulting pink solution was heated at 60 °C for 16 h. The crude product was precipitated by the addition of excess ethyl acetate under vigorous stirring. The pinkish precipitate was purified by centrifugation/redispersion in ethyl acetate (twice, 10 min, 5000 rpm) and dried. Yield: 0.690 g of P(HPMA). For the polymer’s characteristics, see Table 2.

**Figure 5:**
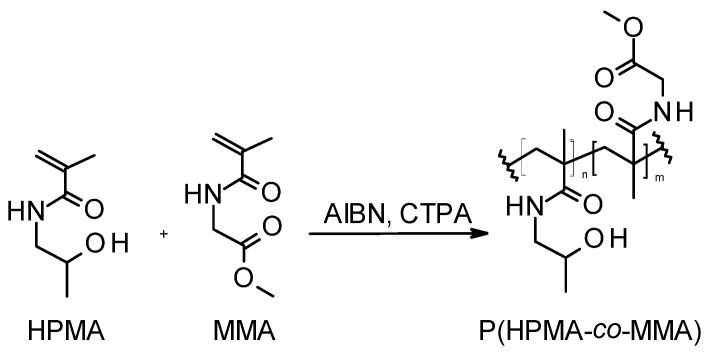
Copolymerization of HPMA and MMA.

Eventually, HPMA (0.9 g, 6.3 mmol) and MMA (0.1 g, 0.70 mmol) were used as comonomers (Figure 5). Yield: 0.675 g of P(HPMA-*co*-MMA). For characterization data, see Table 2.

**Figure 6:**
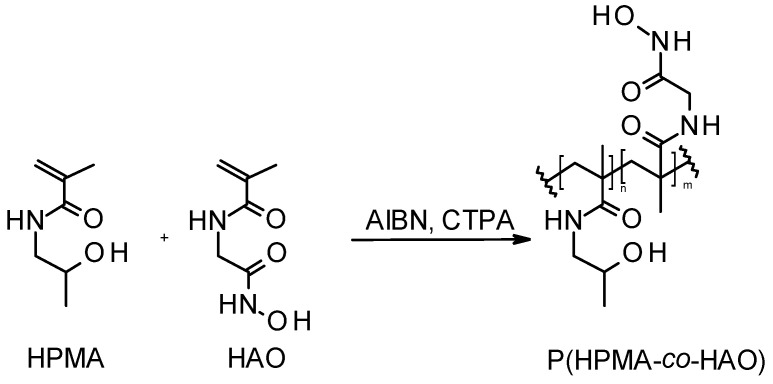
Copolymerization of HPMA and HAO.

The same monomer ratio was utilized for the synthesis of P(HPMA-*co*-HAO) from the appropriate monomers (Figure 6). After precipitation from ethyl acetate, the crude polymer was purified from the unreacted monomer on a Sephadex^®^ G-25 column, firstly in PBS buffer, and then in water. The collected eluate was lyophilized. Yield: 0.360 g of P(HPMA-*co*-HAO). For characterization data, see Table 2.

### 2.6. Functional Modifications of P(HPMA-co-MMA)

#### 2.6.1. Hydrolysis: Synthesis of N-(2-Methyl-1-oxo-1,2-propandiyl)glycine Comonomer Unit (GLM)

**Figure 7:**
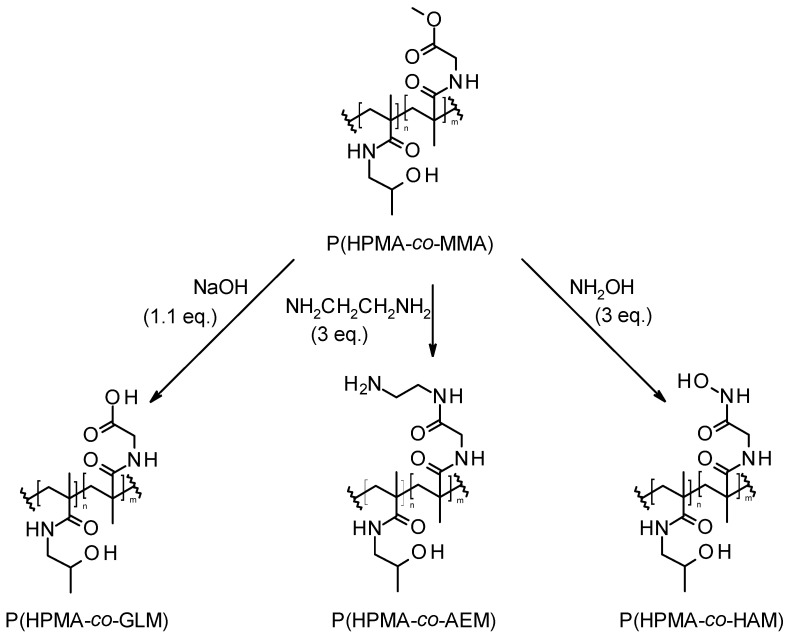
Post-polymerization modifications of the ester group of MMA.

P(HPMA-*co*-MMA) (0.550 g, 0.59 mmol) was introduced into a 100 mL flask and dissolved in ethanol (10 mL). Sodium hydroxide (2 M, 0.34 mL) was added to the mixture and the reaction mixture was stirred for 3 h under an argon atmosphere (Figure 7). The solvent was partially evaporated, and the crude product was purified by Purolite C120 strong acid cation exchanger. The polymer was then precipitated by addition of excess ethyl acetate and purified by centrifugation/redispersion as previously described.

Yield: 0.481 g of P(HPMA-*co*-GLM). For characterization data of P(HPMA-*co*-GLM), see Table 3.

#### 2.6.2. Aminolysis: Synthesis of N-[2-[(2-Aminoethyl)amino]-2-oxoethyl]-2-methyl-1,2-propandiyl-2-amide Comonomer Unit (AEM)

First of all, the copolymer P(HPMA-*co*-MMA) was precipitated from diethylether. P(HPMA-*co*-MMA) (0.550 g, 0.59 mmol) was dissolved in 6 mL of ethanol and added dropwise to a vigorously stirred solution of 1,2-diaminoethane (0.12 mL, 1.8 mmol) in 2 mL of ethanol under an argon atmosphere (Figure 7). The reaction proceeded for 1 h at room temperature. The product was precipitated by adding of an excess of diethyl ether and purified by centrifugation/redispersion, as in the case of its precursor.

Yield: 0.486 g of P(HPMA-*co*-AEM). For characterization data of P(HPMA-*co*-AEM), see Table 3.

#### 2.6.3. Derivatization by Hydroxamic Acid: Synthesis of N-[2-(Hydroxyamino)-2-oxo-ethyl]-2-methyl-1,2-propandiyl-2-amide (HAM)

The copolymer P(HPMA-*co*-MMA) (0.610 g, 0.66 mmol) was dissolved in 10 mL of ethanol, and 0.7 mL of water was added, followed by a 50% water solution of hydroxylamine (0.79 mL, 10.5 mmol) (Figure 7). The yellowish solution was stirred for 16 h at room temperature. The product was precipitated by the addition of excess diethyl ether and purified by centrifugation/redispersion, as in the case of its precursor.

Yield: 0.530 g of P(HPMA-*co*-HAM). For characterization data of P(HPMA-*co*-HAM), see Table 3.

### 2.7. Nuclear Magnetic Resonance Spectroscopy (NMR)

Structures of synthesized monomers were investigated by ^1^H NMR spectroscopy in deuterated solvents on a Bruker DPX spectrometer (Bruker, BioSpin, Ettlingen, Germany) operating at 300.1 MHz. All ^1^H spectra were calibrated to an internal standard of TMS δ = 0.00 ppm.

### 2.8. Elemental Analysis

Elemental analysis was performed on a Perkin Elmer 2400 Series II CHNS/O Elemental Analysis device.

### 2.9. Size Exclusion Chromatography Analysis (SEC)

The number-average molecular weight (*M*_n_) and weight-average molecular weight (*M*_w_), as well as the dispersities *Ð* of the polymers, were determined by size exclusion chromatography on an HPLC system (Shimadzu, Japan) equipped with an internal UV-vis diode array detector (SPD-M20A), external differential refractometer (Optilab T-rEX) and multi-angle light-scattering detector (DAWN HELEOS II, both Wyatt Technology, Santa Barbara, CA, USA). Samples were analyzed on TSKgel SuperAW3000 and SuperAW4000 columns (Tosoh Bioscience, South San Francisco, CA, USA) in a mobile phase of 80% methanol/20% sodium acetate buffer (0.3 M, pH 6.5) at a flow rate of 0.6 mL/min.

### 2.10. Dynamic Light Scattering (DLS)

DLS measurements were performed on a Zetasizer Nano Series ZEN3500 (Malvern, Worcestershire, UK) with a scattering angle of 173° and a 4 mW, 633 nm laser. Hydrodynamic Diameter (*D_h_*), polydispersity index (*PI*; [0; 1]) and zeta potential (*ζ*; calculated by the Smoluchowski method) of the samples were measured in disposable folded DTS1070 capillary cells. Arithmetic means ($\bar{x}$) and corrected standard deviations (*s*) of the means of all three parameters were calculated from three measurements using the following equations:

(1) $$\bar{x}=\frac{1}{N}\sum_{i=1}^{N}x_{i}$$

(2) $$s=\sqrt{\frac{1}{N-1}\sum_{i=1}^{N}{\left(x_{i}-\bar{x}\right)}^{2}.}$$

In a standard experiment, 50 µL of the appropriate colloid (6 mg/mL) and 50 µL of the polymer (3 mg/mL) were introduced into 1 mL of water, PBS buffer or NaCl solution of various concentrations (1 mL, 1 M, 0.5 M, 0.1 M, or 0.01 M). In the case of the experiments with albumin, 50 µL (10 mg/mL) of water solution of albumin was added, and the sample was stirred before the measurement. Measurements of particles coated by the copolymer containing hydroxamic acid groups were performed in a PBS buffer set to appropriate pH (4–10, 1 pH unit step) using aqueous HCl and NaOH solutions.

### 2.11. Transmission Electron Microscopy (TEM)

Transmission electron microscopy was used for the evaluation of nanoparticle size and morphology. Microphotographs of at least 300 nanoparticles per sample were obtained by a transmission electron microscope FEI-TEM, Tecnai G2 Spirit (FEI Company, Hillsboro, OR, USA). ImageJ analysis software, v. 1.53t, was utilized for picture analysis, where diameters of at least 300 nanoparticles (optimally more than 500) were measured manually [70]. Samples for analysis were prepared by depositing diluted aqueous dispersions of nanoparticles on a grid with a copper membrane and carbon film and allowing the samples to dry at room temperature. Number-average diameter (*D_n_*), weight-average diameter (*D_w_*) and dispersity (*Ð*) [71] were calculated from TEM micrographs using equations:

(3) $$D_{n}=\frac{\sum_{i}n_{i}D_{i}}{n_{i}}$$

(4) $$D_{w}=\frac{\sum_{i}n_{i}D_{i}w_{i}}{\sum_{i}n_{i}w_{i}}=\frac{\sum_{i}n_{i}D_{i}V_{i}}{n_{i}\rho V_{i}}=\frac{\sum_{i}n_{i}D_{i}\rho \frac{1}{6}\pi D_{i}^{3}}{\sum_{i}n_{i}\rho \frac{1}{6}\pi D_{i}^{3}}=\frac{\sum_{i}n_{i}D_{i}^{4}}{\sum_{i}n_{i}D_{i}^{3}}$$

(5) $$Ð=\frac{D_{w}}{D_{n}};[1,\infty )$$

where *n_i_* is the number of particles, *D_i_* is the diameter of the nanoparticles, *w* is the mass fraction, *ρ* is the density and *V* is the volume.

### 2.12. Thermogravimetric Analysis (TGA)

TGA was performed in air using a Perkin Elmer TGA 7 analyzer (Norwalk, CT, USA) with 10 °C·minute^−1^ heating step from 30 to 800 °C. In a standard procedure, aqueous solution of P(HPMA-*co*-HAO) (30 mg, 0.8 mL) was added to a magnetic colloid γ-Fe_2_O_3_^⨁/⊖^ (1 mL, 60 mg/mL), and the resulting stabilized colloids were shaken for 15 min. The magnetic particles were separated by repeated (4×) centrifugation/redispersion (4 h, 10,000 rpm) and lyophilized afterwards.

### 2.13. Biodistribution of the Nanoparticles In Vivo

In vivo experiments were performed on C57BL/6NCrl mice bred in a specific-pathogen-free animal facility of the First Faculty of Medicine, Charles University (Prague, Czech Republic), and maintained in individually ventilated cages (12:12 h light–dark cycle, 22 ± 1 °C, 60 ± 5% humidity). The study used adult male mice (6–8 weeks old) with free access to water and a standard rodent diet. The experiments were approved by the Laboratory Animal Care and Use Committee of the First Faculty of Medicine, Charles University, and the Ministry of Education, Youth and Sports of the Czech Republic (MSMT-2309/2018-4). The approved protocol was in accord with the Act of the Czech Parliament for the Protection of Animals Against Cruelty No. 246/1992 and the Directive 2010/63/EU of the European Parliament. Experiments were designed on the principle of the “Three Rs” (replacement, reduction and refinement). All procedures (e.g., contrast agent application and MRI scanning) were performed under anesthesia (passive inhalation of isoflurane in air: 3% for induction, 1.5–2% for maintenance) and the mice were placed on a heated bed to minimize the stress and discomfort of the animals during the measurement and recovery from anesthesia.

A dose of 18 µL of the suspension of nanoparticles (γ-Fe_2_O_3_^⨁/⊖^, γ-Fe_2_O_3_^⨁/⊖^@P(HPMA-*co*-HAO), with a concentration of 4.4 mg/mL of Fe_2_O_3_, i.e., 3.08 mg/mL of Fe) was mixed with 18 µL of saline solution and injected through the tail vein into the experimental mice (animal weight at the time of injection was 16 g). The dose of 55.4 µg Fe/mouse corresponded to 3.47 g Fe/kg of body weight; an equivalent human dosage would be 0.282 mg Fe/kg in humans, when normalized for body surface area [72]. The dosage was within the recommended interval of 0.2–0.8 mg Fe/kg [73] in humans, and did not exceed the FDA-recommended dose of ferucarbotran (0.56 mg Fe/kg). Each nanoparticle type was injected into four mice. The mice were scanned using an ICON MRI scanner (Bruker, Ettlingen, Germany) working at 1 T. The scanning protocol consisted of a fast gradient echo sequence in three anatomical directions for localization (echo time TE = 3 ms, repetition time TR = 107 ms, flip angle FA = 30°), an anatomical gradient echo sequence with mixed T1–T2* weighting (TE = 4 ms, TR = 160 ms, FA = 80°), and a strongly T2*-weighted gradient echo sequence (TE = 8 ms, TR = 400 ms, FA = 60°). The latter two sequences were in a coronal direction; FOV = 50 × 25 mm^2^ covered most of the mouse’s body, and the matrix was 256 × 128.

Scanning was performed before application of the nanoparticles, within 30 min after application (with respect to the length of the measuring protocol) and again 4 h after, and then 1, 3, 7 and 14 days after application. A control group of four mice received the same volume of ferucarbotran (Resovist^®^) [68] suspension diluted to the same iron concentration.

Signal intensity was evaluated in the liver and kidney (medulla and cortex separately). The signal was related to that obtained during control measurement before nanoparticle application.

## 3. Results and Discussion

### 3.1. Synthesis and Coating of the Nanoparticles

In our study we present a modified synthesis of maghemite nanoparticles oxidized by hydrogen peroxide instead of sodium hypochlorite [74], in order to prepare particles stabilized with either a positive or a negative surface charge. Because the total surface charge of the nanoparticles can have a great impact on their hydrodynamic properties [75] and behavior in biological systems [76,77] we investigated their colloidal characteristics. DLS data provide valuable insight into the colloidal behavior of the nanoparticles. Generally, the lower the *D_h_* and *PI*, the more stable the colloid is. In addition, an absolute value of zeta potential above |30| mV indicates a good electrostatic stabilization. The hydrodynamic diameters of both γ-Fe_2_O_3_^⨁^ and γ-Fe_2_O_3_^⊖^ stock colloid samples in water were 96.9 ± 0.9 nm and 96 ± 1, respectively. The polydispersity indices (*PI*) of the novel nanoparticles were 0.153 ± 0.01 for γ-Fe_2_O_3_^⨁^ nanoparticles and 0.17 ± 0.02 for γ-Fe_2_O_3_^⊖^ nanoparticles. Such low *PI* values confirm a narrow size distribution of hydrodynamic sizes; in other words, it confirms the absence of aggregates/agglomerates. Zeta potential values for both washed samples confirm the existence of a very good electrostatic stabilization; in the case of positively charged particles, *ζ* = +41 mV (pH = 4.7) was observed, and for negatively charged particles, *ζ* = –51 mV (pH = 7.3) was observed.

**Figure 8:**
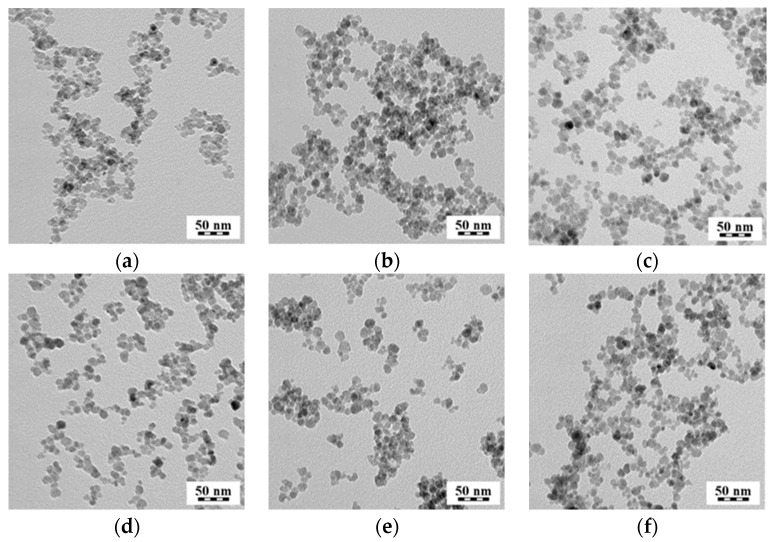
Comparison of TEM micrographs of (**a**) γ-Fe_2_O_3_^⨁^, (**b**) γ-Fe_2_O_3_^⊖^, (**c**) γ-Fe_2_O_3_^⨁^@P(HPMA-*co*-HAO), (**d**) γ-Fe_2_O_3_^⊖^@P(HPMA-*co*-HAO), (**e**) γ-Fe_2_O_3_^⨁^@P(HPMA-*co*-HAM), and (**f**) γ-Fe_2_O_3_^⊖^@P(HPMA-*co*-HAM).

As the DLS provides the information about the hydrodynamic behavior of the magnetic nanoparticles, including their solvation shells (and in case of coated ones also the anchored polymer), their hydrodynamic diameter is significantly higher than their dry size measured by TEM. Another contribution to the difference in *D_h_* (obtained by DLS) and *D_n_* (obtained by TEM) values is the different statistical treatment: while *D_n_* is weighted by the number of particles, *D_h_* represents a *Z* statistical weight that is equivalent to the fraction of the sixth over the fifth powers of the nominal particle diameter. Number average diameters (*D_n_*) of the uncoated nanoparticles obtained by TEM measurements show only slightly higher values (8.0 nm for γ-Fe_2_O_3_^⨁^ and 8.7 nm for γ-Fe_2_O_3_^⊖^) than for the particles oxidized by sodium hypochlorite (6–7 nm) [74]. On the other hand, the *PDI* value (1.3) found for both samples of novel nanoparticles is on the lower border of the published results (1.3–1.5) [74]. These data indicate a narrow size distribution in terms of size; the pictures in Figure 8 prove the spheroid shape of the particles.

The uncoated nanoparticles were subsequently modified with HPMA-based (2-hydroxypropylmethacrylamide) copolymers bearing functional groups (amine, carboxylic acid and hydroxamic acid) in order to sterically stabilize the nanoparticles with a polymer coating exhibiting high affinity to the particles and thus improve their biocompatibility. In particular, hydroxamic acid groups were considered because of their known effective involvement in the iron metabolism. The monomer precursor MMA was prepared by a standard condensation reaction of glycine methyl ester hydrochloride with methacryloyl chloride in a basic solution, and its hydroxamic acid derivate HAO was obtained by the subsequent reaction of MMA with hydroxylamine. The ^1^H NMR spectrum of MMA, measured in CDCl_3_, showed all expected signals for the methacrylic part of the molecule, CH_2_ and an ester group signal. An appropriate number of signals belonging to the acidic protons was also observed. The ^1^H NMR spectrum of HAO was measured in DMSO due to the higher polarity of the compound. Compared to MMA, no methyl ester signal was observed and two new signals due to NH and OH protons appeared, which proved the successful reaction.

Next, we performed RAFT polymerization to obtain polymers with relative molecular weight below the limit of renal filtration, which is usually between 30 and 50 kDa [78], but recent results point to a more complex issue [79]. The polymerization reaction took place in the presence of AIBN as a radical initiator, and dithiobenzoate (CTPA) as chain transfer agent in an acidic solution to produce P(HPMA) and copolymers P(HPMA-*co*-MMA) and P(HPMA-*co*-HAO). According to the SEC measurements, the molecular weight *M*_w_ of the (co)polymers is in the range 39.0–58.3 (kg·mol^−1^) with dispersity (1.19–1.26). The RAFT technique can usually provide polymers with lower dispersity. For the further optimization of the product dispersity the use of the trithiocarbonate type of chain transfer agent instead the dithiobenzoate type can be recommended. Trithiocarbonates exhibit higher hydrolytical stability and cause a lesser retardation rate than dithiobenzoates [80]. Another way to improve the polymer dispersity could be lowering the molar ratio initiator/charge transfer agent (CTA). However, for the aim of this work, the obtained dispersity values are acceptable.

Post-polymerization modification of P(HPMA-*co*-MMA) led to copolymers containing amino, carboxylic, and hydroxamic acid groups randomly distributed along their polymer chains. Molecular weights of the post-synthetically modified copolymers corresponded to the particular modification of the parent polymer (Table 2 and Table 3).

### 3.2. Effect of Coating on Hydrodynamic Stability in Different Liquid Media

The presence and type of the coating polymer had a great impact on the stability and size of the nanoparticles in various media. The observed behavior can be ascribed to a dominant steric stabilization effect provided by the adhered polymer.

**Scheme 1:**
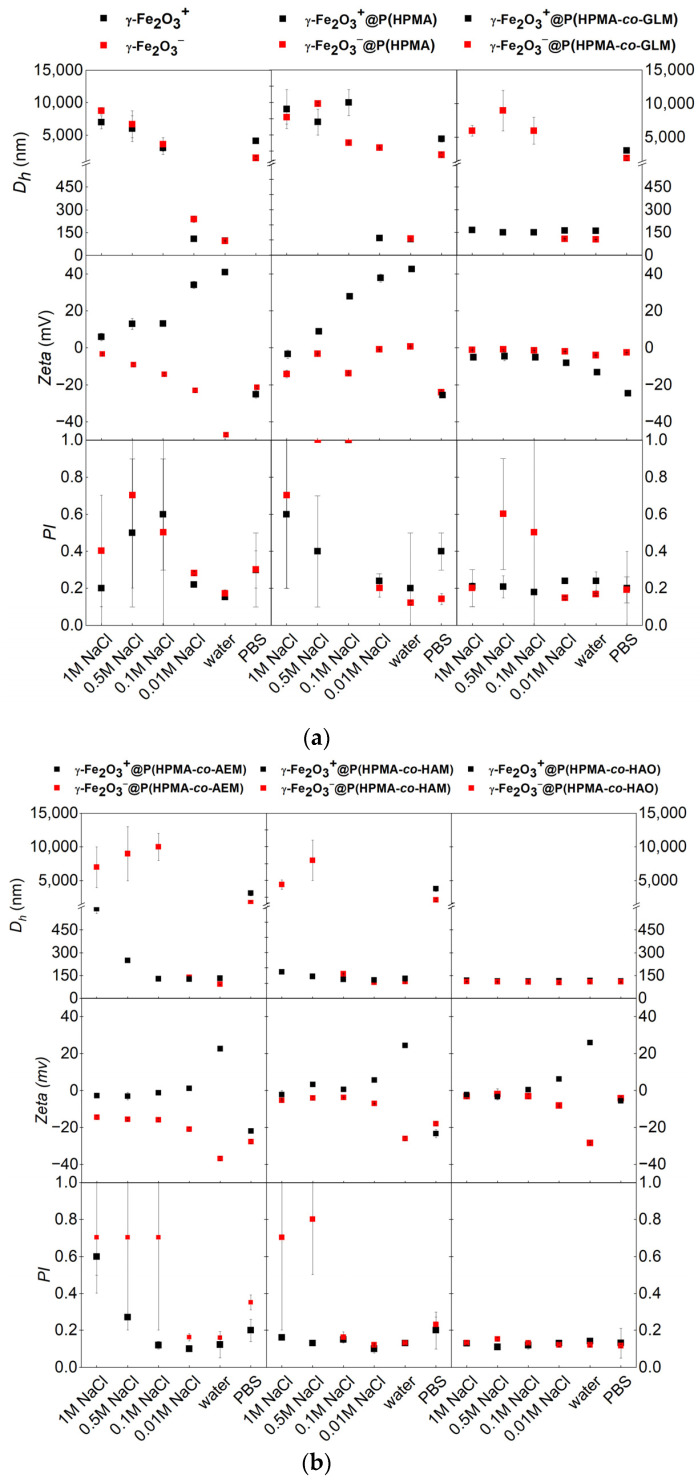
(**a**) DLS monitoring of the behavior of γ-Fe_2_O_3_^⨁/⊖^, γ-Fe_2_O_3_^⨁/⊖^@P(HPMA) and γ-Fe_2_O_3_^⨁/⊖^@P(HPMA-*co*-GLM) colloids at different ionic strengths. (**b**) DLS monitoring of the behavior of γ- γ-Fe_2_O_3_^⨁/⊖^@P(HPMA-*co*-AEM), γ-Fe_2_O_3_^⨁/⊖^@P(HPMA-*co*-HAM) and γ-Fe_2_O_3_^⨁/⊖^@P(HPMA-*co*-HAO) colloids at different ionic strengths.

In the case of polymer absence, neat γ-Fe_2_O_3_^⨁^ and γ-Fe_2_O_3_^⊖^ nanoparticles tend to aggregate, even with a low elevation of ionic strength, which was documented by the increase of the hydrodynamic size (Scheme 1a, Tables S1 and S2 in Supplementary Material). This is also accompanied by an increase of the polydispersity index above 0.2 (it indicates the formation of aggregates/agglomerates) and a proportional decrease of absolute values of zeta-potential to the region around zero, which indicates that the effect of electrostatic stabilization has been significantly counteracted.

Similar behavior (aggregation in a higher ionic strength) was also observed by γ-Fe_2_O_3_^⨁^@P(HPMA), and it was related to the insufficient steric stabilization of the nanoparticles by this polymer because of the lack of the polymer’s affinity to the particle surface (Table S3 in Supplementary Material). In case of γ-Fe_2_O_3_^⊖^@P(HPMA), a small amount of sedimenting precipitate in the colloid is formed, even in water, which is accompanied by zeta-potential rising up to 1 mV, though the *D_h_* and *PI* values remain small (Table S4 in Supplementary Material). The observed partial precipitation can be ascribed to the effect of particles’ bridging being mediated by the added polymer. In the solutions with higher ionic strength, complete aggregation occurred. This behavior can be attributed to some unspecified surface charge compensation on the surface of nanoparticles, which suppresses the already weak adsorption interactions between the polymer and the particles, so that the steric stabilization effect is eliminated [81,82,83].

The polymer coating containing carboxylic- and amino-anchoring functional groups (γ-Fe_2_O_3_^⨁/⊖^@P(HPMA-*co*-GLM) and γ-Fe_2_O_3_^⨁/⊖^@P(HPMA-*co*-AEM)) improved the colloidal stability of nanoparticles only under limited conditions (Scheme 1a,b). Nanoparticles γ-Fe_2_O_3_^⊖^@P(HPMA-*co*-GLM) and γ-Fe_2_O_3_^⨁^@P(HPMA-*co*-AEM) were stable in water and at slightly elevated ionic strength (Tables S6 and S7 in Supplementary Material). In the cases of γ-Fe_2_O_3_^⨁^@P(HPMA-*co*-GLM) and γ-Fe_2_O_3_^⊖^@P(HPMA-*co*-AEM), the presence of the polymer results in the partial precipitation of the mixture, even in the water (Tables S5 and S8 in Supplementary Material), which can be ascribed to compensation of surface charges, as we mentioned above. This behavior documents the fact that neither the carboxylic group nor the amino group is a sufficiently powerful anchoring group for steric stabilization of the maghemite nanoparticles at higher ionic strength. In the presence of electrolyte, the polymers seem to leave the coordination sphere of the maghemite surface iron atoms rapidly.

**Scheme 2:**
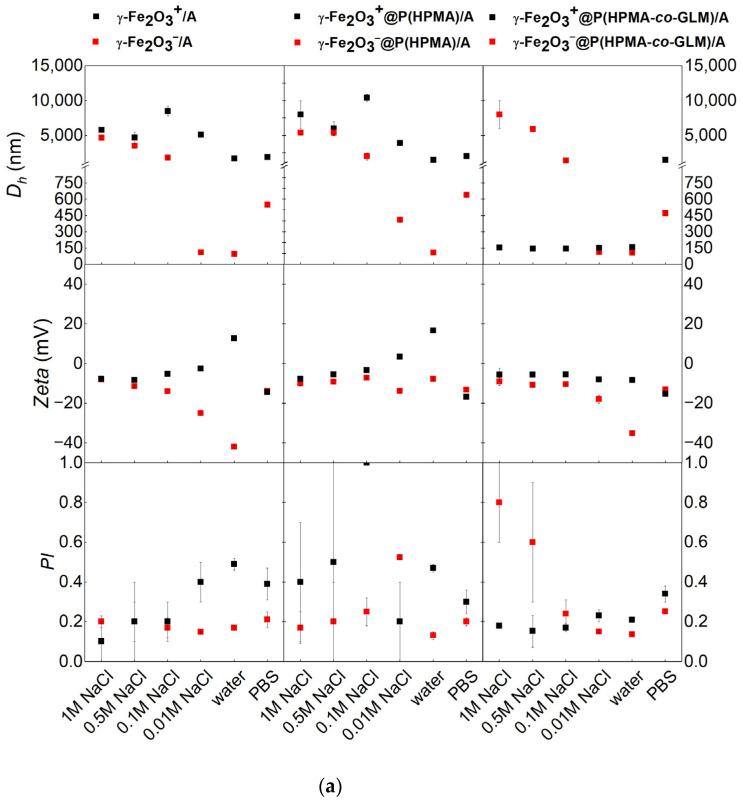

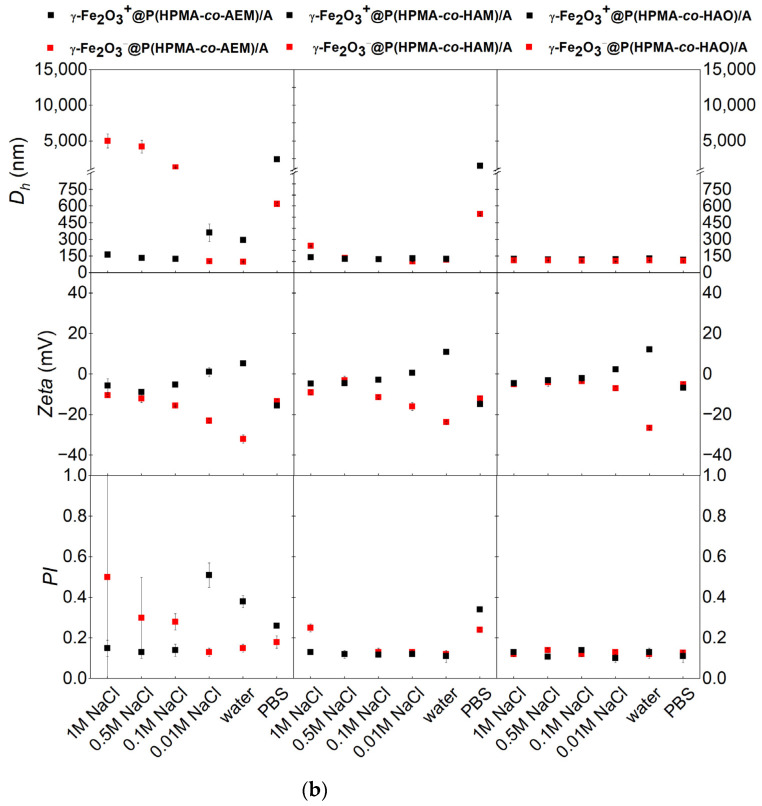
(**a**) DLS data for γ-Fe_2_O_3_^⨁/⊖^, γ-Fe_2_O_3_^⨁/⊖^@P(HPMA), γ-Fe_2_O_3_^⨁/⊖^@P(HPMA-*co*-GLM) colloids in different ionic strengths in the presence of albumin. (**b**) DLS data for γ-Fe_2_O_3_^⨁/⊖^@P(HPMA-*co*-AEM), γ-Fe_2_O_3_^⨁/⊖^@P(HPMA-*co*-HAM) and γ-Fe_2_O_3_^⨁/⊖^@P(HPMA-*co*-HAO) colloids in different ionic strengths in the presence of albumin.

Completely different colloidal behavior in various ionic strengths was observed for the nanoparticles coated by the polymers containing chelating hydroxamic acid groups (entry γ-Fe_2_O_3_^⨁/⊖^@P(HPMA-*co*-HAM) and γ-Fe_2_O_3_^⨁/⊖^@P(HPMA-*co*-HAO)) (Scheme 1b, Tables S9–S12, in Supplementary Material). Coated particles were stable, not only in ultrapure water, but also in electrolyte solutions with high ionic strength. Nanoparticles coated by both kinds of hydroxamate functionalized polymer (entry γ-Fe_2_O_3_^⨁^@P(HPMA-*co*-HAM) and γ-Fe_2_O_3_^⨁/⊖^@P(HPMA-*co*-HAO)) formed very stable colloids with the hydrodynamic size not exceeding 130 nm for the positively charged and 115 nm for the negatively charged coated nanoparticles (Tables S9, S11 and S12 in Supplementary Material). The only exception is represented by γ-Fe_2_O_3_^⊖^@P(HPMA-*co*-HAM), which formed stable colloid in the water, but partially precipitated at higher ionic strength (Table S10 in Supplementary Material). This behavior resembles that of the previously mentioned γ-Fe_2_O_3_^⨁^@P(HPMA-*co*-GLM) and γ-Fe_2_O_3_^⊖^@P(HPMA-*co*-AEM). Zeta-potential (of all four kinds of nanoparticles coated by copolymer containing hydroxamic acid groups) at higher ionic strength decreased to values near zero (due to a charge compensation), which means that the electrostatic stabilization is inefficient. Nevertheless, due to the strong steric stabilization of the particles by the layer of polymers, the coated nanoparticles remained in a colloidal state, with no evidence of aggregation. Surprisingly, for all four kinds of nanoparticles coated by copolymer containing hydroxamic acid groups, the zeta-potential in ultrapure water systematically reached values around |22|–|29| mV. This behavior documents the character of the interaction between the polymer and nanoparticles, which is dominantly based on coordination instead of ionic interactions (as in case of amino- and carboxyl-functionalized polymers) (Scheme 2b, Tables S9–S12 in Supplementary Material). A higher tendency for aggregation (insufficient coating potential of the HAM copolymer, especially for negatively charged nanoparticles) probably corresponds to the dissimilarity of the synthesis of the polymers. While polymerization of HPMA with comonomer containing the hydroxamic acid group produced copolymer with excellent coating capability, post-synthetic modification led to slightly worse results. The reason can be found in the mildly lower concentration of hydroxamic acid groups in the post-synthetically modified copolymer due to incomplete conversion. A different architecture of the polymer with a lower amount of the anchoring groups might lead to possible bridging between particles and a subsequent higher tendency for aggregation. Nanoparticles γ-Fe_2_O_3_^⨁/⊖^@P(HPMA-*co*-HAO), which were stable even in solutions with the highest tested ionic strength (1 M NaCl), seem to have a higher density of the hydroxamic acid anchoring groups in close proximity to the surface of the particles, with a lower portion of floating segments, which in turn enhances its steric stabilization potential. Since the copolymerization with an unprotected monomer containing hydroxamic-acid-group did not display any unwanted effects (e.g., inhibition transfer) during the polymerization, it can be concluded that direct copolymerization of hydroxamate monomer is an easier and more effective strategy for synthesis of this type of copolymers in comparison with post-polymerization modification.

In addition to the study of ionic strength changes in the presence of NaCl, evaluation of behavior in the PBS buffer was performed. The presence of PBS led to aggregation in all cases except for γ-Fe_2_O_3_^⨁/⊖^@P(HPMA-*co*-HAO) (Scheme 1a,b, Tables S11 and S12 in Supplementary Material). The PBS buffer changes the ionic strength, but in contrast to NaCl solution, phosphate ions also interact with the nanoparticles, forming iron phosphate complexes. This interaction was strong enough to change the coordination spheres of the uncoated as well as the coated nanoparticles where the anchoring group wasn’t present or was not bound strongly enough to persist on the surface. All of these nanoparticles aggregated in the same manner. In contrast with these nanoparticles, γ-Fe_2_O_3_^⨁/⊖^@P(HPMA-*co*-HAO) kept colloidal stability, even in the PBS buffer, probably because of the chelating effect of the hydroxamate anchoring groups mentioned above.

In order to investigate the coating and stabilization efficiency of the polymer coatings, an addition of albumin was applied to investigate the effect of non-specific protein adsorption on the uncoated and coated nanoparticles, and their behavior in the presence of protein in relation to the coating type. Its dose (equal volume, 10 mg/mL) led to immediate aggregation of neat γ-Fe_2_O_3_^⨁^ and γ-Fe_2_O_3_^⨁^@P(HPMA) in all liquid systems regardless of the ionic strength (Scheme 2a, Tables S1 and S3 in Supplementary Material). This behavior can be explained by the fact that positive surfaces generally induce nonspecific adsorption of proteins [84]. At the physiological pH, the albumin molecule is negatively charged (the isoelectric point of albumin varies from 4.7 to 4.9) and, therefore, it is more attracted to the magnetic particles with a positive charge. The colloidal behaviors of γ-Fe_2_O_3_^⊖^, γ-Fe_2_O_3_^⊖^@P(HPMA), γ-Fe_2_O_3_^⨁/⊖^@P(HPMA-*co*-GLM) and γ-Fe_2_O_3_^⊖^@P(HPMA-*co*-AEM) seem to be unaffected by albumin presence in the water solution, and the absolute values of the *PI* remain low (Scheme 2a,b, Tables S2, S4–S6 and S8 in Supplementary Material). In the presence of albumin, nanoparticles γ-Fe_2_O_3_^⨁^@P(HPMA-*co*-AEM) precipitated in water and 0.01 M NaCl; nevertheless, in higher ionic strengths they formed a stable colloid or a colloid with small portion of precipitate (Table S7).

Nanoparticles γ-Fe_2_O_3_^⨁/⊖^@P(HPMA-*co*-HAM) displayed reasonably good colloidal stability after addition of albumin, in wide range of ionic strength (Scheme 2b, Tables S9 and S10). The best stabilization of the maghemite nanoparticles in the presence of albumin was provided by the polymer P(HPMA-*co*-HAO) (entry γ-Fe_2_O_3_^⨁/⊖^@P(HPMA-*co*-HAO), (Scheme 2b, Tables S11 and S12 in Supplementary Material). Low *D_h_* and *PI* prove colloidal stability in the whole tested range of ionic strengths. This behavior proves the fact that the chelating effect of the anchoring hydroxamic acid groups determines the high stability of the coating, which is not disturbed by ionic or complex interaction or weak non-polar interactions with proteins.

### 3.3. Evaluation of the Size and Shape Changes of the (Un)Coated Nanoparticles by TEM Measurement

As mentioned above, DLS provides information about magnetic nanoparticles, together with their polymer coating and aqueous solvation shell. At the available accelerating voltage (100 eV), TEM shows only the core of the magnetic nanoparticles due to the low contrast of the polymer carbons compared to that of the much heavier maghemite iron atoms (if a higher voltage was used it might have been possible to see the coating polymer on the particles). TEM images of the novel nanoparticles revealed high uniformity in terms of a narrow size distribution and a spheroid shape. Schemes S1–S3 in the Supplementary Material display the narrow size distribution for γ-Fe_2_O_3_^⨁/⊖^ as well as for selected coated particles which remained colloidally stable at given conditions. The size of the particles did not significantly increase after coating; thus, no aggregation or sintering occurred, and no erosion of the nanoparticles was observed. Figure 8 documents the shape and size of selected particles before and after coating. For comparison, TEM analysis was also performed for the nanoparticles (coated by the copolymer containing hydroxamic acid groups) in the presence of albumin. The core size of the particles did not change significantly.

### 3.4. TGA Analysis

**Scheme 3:**
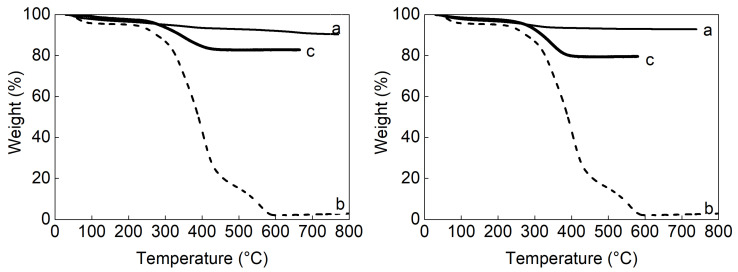
TGA data for (**left**) γ-Fe_2_O_3_^⨁^ (a), P(HPMA-*co*-HAO) (b) and γ-Fe_2_O_3_^⨁^@P(HPMA-*co*-HAO) (c); (**right**) γ-Fe_2_O_3_^⊖^ (a), P(HPMA-*co*-HAO) (b) and γ-Fe_2_O_3_^⊖^@P(HPMA-*co*-HAO) (c).

For better understanding of the polymer vs. nanoparticles binding ratio in the case of γ-Fe_2_O_3_^⨁/⊖^@P(HPMA-*co*-HAO) nanoparticles, we have decided to include TGA analysis. TGA analysis was performed for uncoated particles γ-Fe_2_O_3_^⨁/⊖^, polymer containing hydroxamic acid groups P(HPMA-*co*-HAO) and coated nanoparticles γ-Fe_2_O_3_^⨁/⊖^@P(HPMA-*co*-HAO) (Table 4, Scheme 3). The uncoated γ-Fe_2_O_3_^⨁^ burned from 9.64%, and γ-Fe_2_O_3_^⊖^ lost 7.21% of mass after heating to 800 °C. This loss can be attributed to the occluded water. Pure polymer P(HPMA-*co*-HAO) displayed nearly complete combustion (97.75%). The percentage of the polymer bound to the particles was calculated from the TGA analysis. For γ-Fe_2_O_3_^⨁^@P(HPMA-*co*-HAO), the 17.2% of the flammable portion corresponds to 91% of nanoparticle cores and 9% of the polymer. A slightly higher amount of polymer seems to be present on the surface of γ-Fe_2_O_3_^⊖^@P(HPMA-*co*-HAO) (20.50% weight loss) which means that the sample consists of 88% of nanoparticles cores and 12% of polymer. When we consider that the initial loading of the polymer was one-third of the total amount of coated particles, the amount of firmly bound polymer can be easily calculated as 26% of the present polymer in the case of γ-Fe_2_O_3_^⨁^@P(HPMA-*co*-HAO), and 38% of the added polymer for γ-Fe_2_O_3_^⊖^@P(HPMA-*co*-HAO). These data demonstrate the fact that the good stability of the colloid in the large range of salinity and pH, as well as the suppression of unspecified sorption of albumin are not influenced by the amount of the coating but rather the degree of its quality. The results also indicate that, in the case of the copolymer P(HPMA-*co*-HAO), the surface charge of the maghemite cores did not have a significant influence on either the quantity (TGA) or the quality (DLS) of the coating.

### 3.5. Study of the Influence of pH Changes on the Colloidal Behavior of the Selected (Un)Coated Particles

**Scheme 4:**
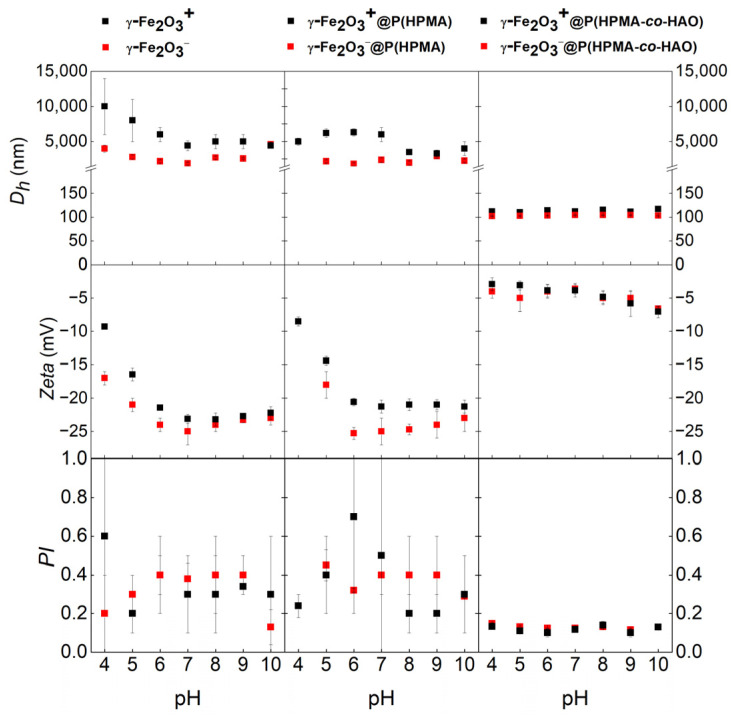
DLS data for uncoated, P(HPMA)- and P(HPMA-*co*-HAO)-coated γ-Fe_2_O_3_^⨁^ and γ-Fe_2_O_3_^⊖^ nanoparticles in PBS buffer, measured at various values of pH.

Moreover, the behavior of the selected nanoparticles was tested at different pH values, in order to complete the investigation of the stability of the particles (Scheme 4, Tables S13 and S14 in Supplementary Material). Uncoated nanoparticles (γ-Fe_2_O_3_^⨁/⊖^) and nanoparticles coated by HPMA (γ-Fe_2_O_3_^⨁/⊖^@P(HPMA) and by the copolymer containing hydroxamic acid units (γ-Fe_2_O_3_^⨁/⊖^@P(HPMA-*co*-HAO)) were chosen for this study. γ-Fe_2_O_3_^⨁/⊖^@P(HPMA-*co*-HAO) were chosen because of their best colloidal stability. The measurements were performed over a pH 4–10 range in a PBS buffer. Immediate aggregation occurred in the cases of uncoated nanoparticles and those coated by P(HPMA), irrespective of pH, for all of the tested samples. This behavior demonstrated the fact that the aggregation was initiated by the addition of PBS and not because of a pH change (this can be observed also in Scheme 1a). The only difference could be seen in zeta-potential change, but it didn’t affect aggregation. The process was accompanied by increase of *PI*. Moreover, values of zeta-potential were found outside of the stability region; all nanoparticle samples (γ-Fe_2_O_3_^⨁/⊖^ and γ-Fe_2_O_3_^⨁/⊖^@P(HPMA) were measured to have negative zeta-potentials (–9 mV to –25 mV), even for originally positively charged nanoparticles. This instability is in good agreement with the assumption of the ligand exchange in the excess of the hydrogen phosphate ions in the solution. On the other hand, the γ-Fe_2_O_3_^⨁/⊖^@P(HPMA-*co*-HAO) particles produced colloids in the entire tested range of pH (4–10), with low polydispersity (Scheme 4, Tables S13 and S14 in Supplementary Material). Phosphate interacts with the surface of these coated nanoparticles, which can be demonstrated by the values of zeta-potential (in the range 3–8 mV). This relatively low absolute value indicates the significant reduction of electrostatic repulsive interaction between the coated nanoparticles.. Nevertheless, due to the coating, the steric stabilization is strong enough that the interaction doesn’t have any influence on the hydrodynamic size and dispersity. This conclusion clearly follows from the comparison of the (un)coated nanoparticles (Scheme 4). This behavior suggests that the hydroxamic acid groups are bound to the iron cations in a manner strong enough that even an excess of phosphate ligand doesn’t lead to change in the coordination sphere.

### 3.6. In Vivo Study

**Figure 9:**
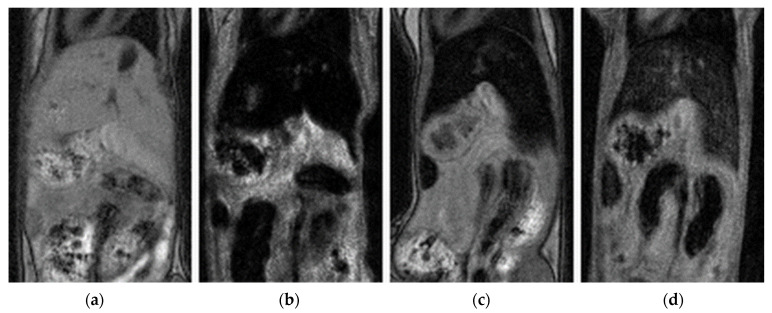
In vivo MRI scans (gradient echo sequence with a mixed T1/T2* contrast) of the liver before nanoparticle (γ-Fe_2_O_3_^⨁^) application (**a**), immediately afterward (**b**), one day afterward (**c**), and 2 weeks afterward (**d**).

The in vivo experiment was aimed at monitoring biodistribution of the nanoparticles after intravenous injection, and their persistence in the tissue (Figure 9).

**Scheme 5:**
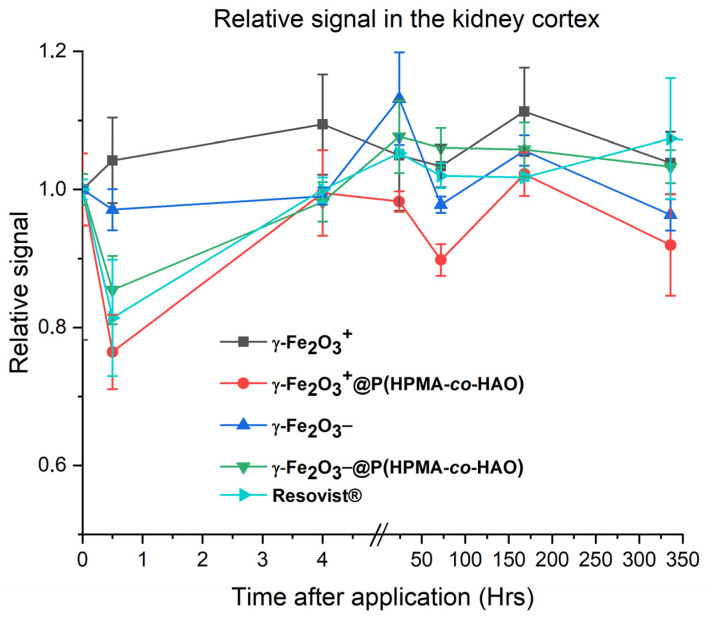
Signal evolution in the kidney cortex after intravenous application of the nanoparticles.

**Scheme 6:**
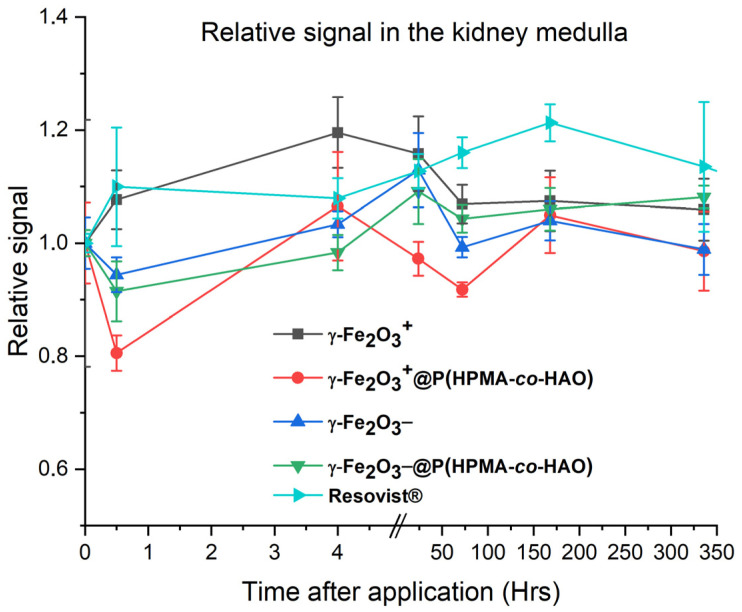
Signal evolution in the kidney medulla after intravenous application of the nanoparticles.

**Scheme 7:**
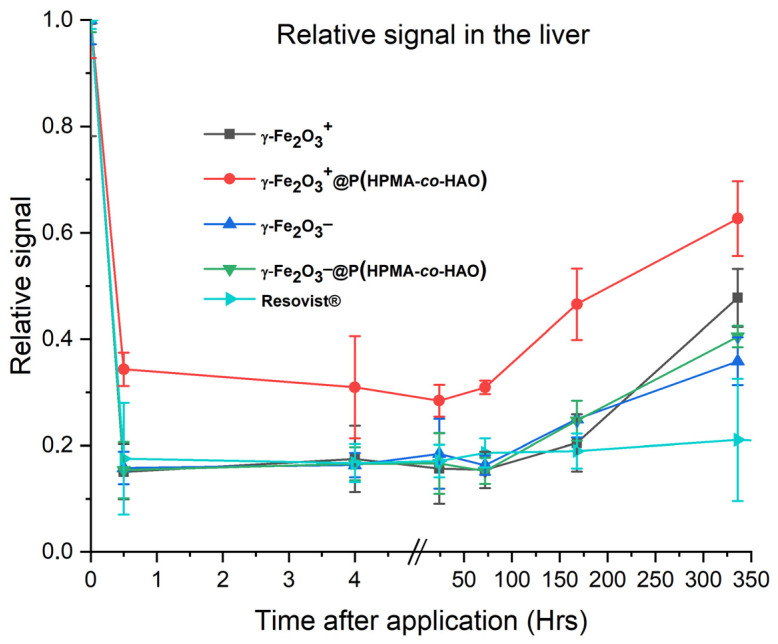
MR signal intensity in the liver corresponding to the clearance of the nanoparticles from the liver tissue.

MRI revealed that the nanoparticles were detectable in highly vascularized organs (i.e., a hypointense signal in the liver, spleen and kidneys) showing their circulation in the blood shortly after application. The particles were cleared from the blood within the first four hours, as manifested by normalization of the signal in the kidneys, see Scheme 5, Scheme 6 and Scheme 7. For detailed data, see Tables S15 and S16 and Figure S1 in the Supplementary Material.

The nanoparticles were entrapped predominantly in the liver tissue, through which they should have been eliminated. Nevertheless, clearance of the particles from the liver was unexpectedly very slow (Scheme 7; detailed data are available in Table S17 in the Supplementary Material). Interestingly, the clearance was not exponential, but rather linear. We speculate that clearance is not driven solely by the nanoparticle concentration in the tissue (which would show exponential behavior). Linear washing out indicates that the used concentration probably saturated the clearance capacity of the tissue, and that we observed the maximum ability for nanoparticle elimination. Most of the tested nanoparticles showed similar time dependence and similar slope. Additionally, part of the iron could have been processed/deposited by the liver tissue as an iron reserve for future use.

The half-life of the nanoparticles in the liver varied from 28 days (γ-Fe_2_O_3_^⨁^@P(HPMA-*co*-HAO)) to 59 days (γ-Fe_2_O_3_^⊖^); see Table 5. The values seem to be extremely high for future clinical applications. However, clearance of the Resovist^®^ (used as a reference in our experiment) was many times longer (Table 5); its clearance half-time was 270 days. Reports on ferucarbotran in clinical practice state that the half-life is much shorter (7–14 days) [85,86]. Our observation indicated possible saturation of the liver’s ability to eliminate the nanoparticles and a substantial slowdown of their clearance. This is interesting in view of the fact that the normalized dose was well below the FDA-recommended dosage for ferucarbotran in clinical practice. While we have no unequivocal explanation of this observation, we cannot exclude differences in the mouse and human metabolisms. In such a case, the usage of a mouse model in preclinical experiments should be approached with caution [87]. Nevertheless, faster elimination of the nanoparticles (compared to Resovist^®^) represents a rather positive contribution.

Nanoparticles γ-Fe_2_O_3_^⨁^ and γ-Fe_2_O_3_^⨁^@P(HPMA-*co*-HAO), whose half-life substantially differed, were stabilized by acid; therefore, they have a positive charge.

## 4. Conclusions

This study confirms the convenient feasibility of the peroxide oxidation of magnetite nanoparticles, which enables the preparation of positively as well as negatively charged particles in the shape of spheroids of ~8–9 nm in diameter, with very similar morphological properties. Copolymers were synthesized via a RAFT copolymerization procedure either directly using HPMA and HAO (comonomer containing hydroxamic acid groups) or by copolymerization of HPMA with MMA, followed by post-polymerization modification (i.e., introduction of amine, carboxylic acid and hydroxamic acid functional groups). The *M_w_* of the copolymers was in the range 39–58 kg·mol^−1^, with *Ð* not exceeding 1.26. These modified copolymers were used for coating maghemite nanoparticles, and the resulting coated particles were studied by TEM and DLS. Nanoparticles γ-Fe_2_O_3_^⊖^@P(HPMA-*co*-HAO) coated by the copolymer synthesized by direct copolymerization and containing hydroxamic acid anchoring groups displayed substantial colloidal stability across the whole range of tested conditions (i.e., high ionic strength, presence of PBS buffer and addition of BSA protein), and over the pH range 4–10. This behavior proves the assumption of high ligand affinity of the hydroxamic acid groups to the presented nanoparticles. Copolymers containing post-polymerization modified anchoring groups (amine, carboxylic acid and hydroxamic acid) provided the nanoparticles with lower colloidal stability, and not even the presence of the hydroxamic acid group in the post-polymerization modified polymer led to results comparable with those of the directly copolymerized one. Direct copolymerization proved to be a more effective way to synthesize the copolymer containing hydroxamic acid groups compared to the post-polymerization modification, probably because of the higher number of functional groups, the different architecture and the consequently enhanced interaction of the polymer with the surface of the nanoparticles. TGA analysis of γ-Fe_2_O_3_^⊖^@P(HPMA-*co*-HAO) indicated that 26 wt. % of the copolymer in the case of positively charged nanoparticles, and 38 wt. % of the copolymer in case of negatively charged nanoparticles, was firmly bound to the surface of the nanoparticles. This amount was sufficient to ensure substantial colloidal stability in various conditions. In vivo experiments on mice confirmed fast clearance of the nanoparticles from the blood flow and their retention mainly in the liver, which is responsible for nanoparticle elimination from the body. While the half-life of the particles seems to be very long (28–38 days for cationic particles and 47–59 days for anionic ones), it is much shorter than commercially available ferucarbotran (Resovist^®^), and therefore, probably much safer for use in vivo.

## Figures and Tables

**Table 1:** Characterization of maghemite particles.

Compound	DLS			TEM			pH
	*D_h_ (nm)*	*PI*	*Zeta (mV)*	*D_n_ (nm)*	*D_w_ (nm)*	*PDI*	
γ-Fe_2_O_3_^⨁^	96.9 ± 0.9	0.153 ± 0.01	+41	8.0	10.0	1.3	3.9
γ-Fe_2_O_3_^⊖^	96 ± 1	0.17 ± 0.02	−51	8.7	11.1	1.3	7.4

**Table 2:** Characterization of P(HPMA), P(HPMA-*co*-MMA) and P(HPMA-*co*-HAO).

Compound	*M_n_* (kg·mol^−1^)	*M_w_* (kg·mol^−1^)	*Ð*
P(HPMA)	37.6	44.7	1.19
P(HPMA-*co*-MMA)	40.6	48.7	1.20
P(HPMA-*co*-HAO)	32.3	40.6	1.26

**Table 3:** Characterization data for P(HPMA-*co*-GLM), P(HPMA-*co*-AEM) and P(HPMA-*co*-HAM).

Compound	*M_n_* (kg·mol^−1^)	*M_w_* (kg·mol^−1^)	*Ð*
P(HPMA-*co*-GLM)	32.2	39.0	1.21
P(HPMA-*co*-AEM)	47.9	58.3	1.22
P(HPMA-*co*-HAM)	39.9	48.1	1.21

**Table 4:** TGA measurement of γ-Fe_2_O_3_^⨁/⊖^, P(HPMA-*co*-HAO) and γ-Fe_2_O_3_^⨁/⊖^@P(HPMA-*co*-HAO).

Compound	Burned Mass (%)	Nanoparticles (%)	Polymer Bound to the Particles (%)	Portion of Bound Polymer Related to the Mass Used for Coating
γ-Fe_2_O_3_^⨁^	9.64	–	–	–
γ-Fe_2_O_3_^⊖^	7.21	–	–	–
P(HPMA-*co*-HAO)	97.75	–	–	–
γ-Fe_2_O_3_^⨁^@P(HPMA-*co*-HAO)	17.2	91	9	26
γ-Fe_2_O_3_^⊖^@P(HPMA-*co*-HAO)	20.50	88	12	38

**Table 5:** Clearance half-times of the nanoparticles from the liver tissue.

Nanoparticles	Clearance Half-Time (Days)
γ-Fe_2_O_3_^⨁^	38 ± 8
γ-Fe_2_O_3_^⊖^	59 ± 36
γ-Fe_2_O_3_^⨁^@P(HPMA-*co*-HAO)	28 ± 13
γ-Fe_2_O_3_^⊖^@P(HPMA-*co*-HAO)	47 ± 43
Resovist^®^	272 ± 150

## Data Availability

Not applicable.

## Supplementary Materials

The following Supplementary Material can be downloaded at: https://www.mdpi.com/article/10.3390/pharmaceutics15071982/s1. Table S1: Behavior of uncoated magnetic nanoparticles (γ-Fe_2_O_3_^⨁^) in various conditions. Table S2: Behavior of uncoated magnetic nanoparticles (γ-Fe_2_O_3_^⊖^) in various conditions. Table S3: Behavior of γ-Fe2O3^⨁^@P(HPMA) in various conditions. Table S4: Behavior of γ-Fe_2_O_3_^⊖^@P(HPMA) in various conditions. Table S5: Behavior of γ-Fe_2_O_3_^⨁^@P(HPMA-*co*-GLM) in various conditions. Table S6: Behavior of γ-Fe_2_O_3_^⊖^@P(HPMA-*co*-GLM) in various conditions. Table S7: Behavior of γ-Fe_2_O_3_^⨁^@P(HPMA-*co*-AEM) in various conditions. Table S8: Behavior of γ-Fe_2_O_3_^⊖^@P(HPMA-*co*-AEM) in various conditions. Table S9: Behavior of γ-Fe_2_O_3_^⨁^@P(HPMA-*co*-HAM) in various conditions. Table S10: Behavior of γ-Fe_2_O_3_^⊖^@P(HPMA-*co*-HAM) in various conditions. Table S11: Behavior of γ-Fe_2_O_3_^⨁^@P(HPMA-*co*-HAO) in various conditions. Table S12: Behavior of γ-Fe_2_O_3_^⊖^@P(HPMA-*co*-HAO) in various conditions. Table S13: Behavior study of selected particles according to pH changes. Table S14: Behavior study of selected particles according to pH changes. Scheme S1. Diameter changes of the magnetic particles coated by P(HPMA-*co*-HAO) depending on different conditions. Scheme S2. Diameter changes of the magnetic particles coated by P(HPMA-*co*-HAM) and P(HPMA-*co*-AEM) depending on different conditions. Scheme S3. Diameter changes of the magnetic particles coated by P(HPMA-*co*-GLM) and P(HPMA) depending on different conditions. Table S15: Relative MR signal changes in the kidney cortex. Table S16: Relative MR signal changes in the kidney medulla. Table S17: Relative MR signal changes in the liver. Figure S1: In vivo MRI scans (gradient echo sequence with a mixed T1/T2* contrast) of the kidney before nanoparticle (γ-Fe_2_O_3_^⨁^) application (**a**), immediately after (**b**), one day after (**c**), and 2 weeks after application (**d**).
