# Supplementary material for: Hydrophilic Copolymers with Hydroxamic Acid Groups as a Protective Biocompatible Coating of Maghemite Nanoparticles: Synthesis, Physico-Chemical Characterization and MRI Biodistribution Study
Source: Pharmaceutics. 2023 Jul 19;15(7):1982. doi: 10.3390/pharmaceutics15071982 (PMC10384990; doi:10.3390/pharmaceutics15071982)
Supplement: Supplementary file 1 [file pharmaceutics-15-01982-s001.zip › pharmaceutics-2474724-supplementary.pdf]

Supplementary Material

Hydrophilic Copolymers with Hydroxamic Acid Groups as a Protective Biocompatible Coating of Maghemite Nanoparticles: Synthesis, Physico-Chemical Characterization and MRI Biodistribution Study

Hana Charvátová [1], Zdeněk Plichta [1], Jiřina Hromádková [1], Vít Herynek [2] and Michal Babič [1,*]

[1] Institute of Macromolecular Chemistry, Czech Academy of Sciences, Heyrovského Náměstí 2, 162 06 Prague, Czech Republic; charvatova@imc.cas.cz (H.C.)
[2] Center for Advanced Preclinical Imaging (CAPI), First Faculty of Medicine, Charles University, Salmovská 3, 120 00 Prague, Czech Republic; vit.herynek@lf1.cuni.cz
* Correspondence: babic@imc.cas.cz

Table S1: Behavior of uncoated magnetic nanoparticles (γ-$Fe_2O_3^{\oplus}$) in various conditions.

Conditions	Albumin	D_h (nm)	*PI*	ζ (mV)	pH	Description of the behavior
1M NaCl	–	7000±1000	0.2±0.2	6±2	5.0	Precipitate
0.5M NaCl	–	6000±2000	0.5±0.4	13±3	4.9	Precipitate
0.1M NaCl	–	3000±1000	0.6±0.3	13.2±0.9	5.0	Precipitate
0.01M NaCl	–	108.8±0.6	0.22±0.01	34±2	5.0	Colloid + precipitate
Water	–	96.9±0.9	0.153±0.01	41±1	4.7	Colloid
PBS buffer	–	4100±400	0.3±0.2	–25±2	7.4	Precipitate
1M NaCl	Albumin	5800±200	0.1±0.1	–7±0.5	5.9	Precipitate
0.5M NaCl	Albumin	4700±800	0.2±0.2	–7.4±0.8	6.7	Precipitate
0.1M NaCl	Albumin	8500±700	0.2±0.1	–4.7±0.7	6.0	Precipitate
0.01M NaCl	Albumin	5100±200	0.4±0.1	–2.31±0.09	5.8	Precipitate
Water	Albumin	1670±5	0.49±0.03	11.5±0.4	5.5	Precipitate
PBS buffer	Albumin	1900±200	0.39±0.08	–13±1	7.4	Precipitate

Table S2: Behavior of uncoated magnetic nanoparticles (γ-$Fe_2O_3^{\ominus}$) in various conditions.

Conditions	Albumin	D_h (nm)	*PI*	ζ (mV)	pH	Description of the behavior
1M NaCl	–	9000±500	0.4±0.3	–8.4±0.5	7.4	Precipitate
0.5M NaCl	–	7000±2000	0.7±0.5	–14±1	7.4	Precipitate
0.1M NaCl	–	4000±1000	0.5±0.5	–19.2±0.7	7.3	Precipitate
0.01M NaCl	–	240±20	0.28±0.01	–28±1	7.5	Precipitate
Water	–	96±1	0.17±0.02	–51±1	7.3	Colloid
PBS buffer	–	2000±200	0.3±0.1	–26.3±0.7	7.4	Precipitate
1M NaCl	Albumin	4700±200	0.20±0.03	–8±0.3	6.4	Precipitate
0.5M NaCl	Albumin	3500±300	0.2±0.1	–11.4±0.5	6.9	Precipitate
0.1M NaCl	Albumin	1790±20	0.17±0.05	–14±1	7.1	Precipitate
0.01M NaCl	Albumin	111.5±0.2	0.148±0.002	–25±1	7.2	Colloid
Water	Albumin	96.1±0.5	0.169±0.009	–41.9±0.6	7.0	Colloid
PBS buffer	Albumin	550±10	0.21±0.04	–13.8±0.5	7.5	Precipitate

Table S3: Behavior of γ-$Fe_2O_3^{\oplus}$@P(HPMA) in various conditions.

Conditions	Albumin	D_h (nm)	*PI*	ζ (mV)	pH	Description of the behavior
1M NaCl	–	9000±3000	0.6±0.4	–3±2	5.8	Precipitate
0.5M NaCl	–	7000±2000	0.4±0.3	8±1	5.1	Precipitate
0.1M NaCl	–	10000±2000	1	25±1	4.7	Precipitate
0.01M NaCl	–	112±2	0.24±0.04	34±2	4.6	Colloid + precipitate
Water	–	105±2	0.2±0.3	38.4±0.6	4.7	Colloid
PBS buffer	–	4400±500	0.4±0.1	–23±1	7.4	Precipitate
1M NaCl	Albumin	8000±2000	0.4±0.3	–7±1	6.2	Precipitate
0.5M NaCl	Albumin	6000±1000	0.5±0.5	–4.9±0.5	5.5	Precipitate
0.1M NaCl	Albumin	10400±500	1	–3±0.1	5.4	Precipitate
0.01M NaCl	Albumin	3900±200	0.2±0.2	3.1±0.5	5.1	Precipitate
Water	Albumin	1500±100	0.47±0.02	15±0.4	5.2	Precipitate
PBS buffer	Albumin	2000±100	0.3±0.06	–15.2±0.9	7.4	Precipitate

Table S4: Behavior of γ-$Fe_2O_3^{\ominus}$@P(HPMA) in various conditions.

Conditions	Albumin	D_h (nm)	*PI*	ζ (mV)	pH	Description of the behavior
1M NaCl	–	8000±1000	0.706±0.5	–14±2	6.7	Precipitate
0.5M NaCl	–	10000±200	1	–3.2±0.3	6.8	Precipitate
0.1M NaCl	–	4200±130	1	–13.9±0.5	7.0	Precipitate
0.01M NaCl	–	3460±80	0.2±0.05	–0.8±0.1	7.0	Precipitate
Water	–	108.4±0.2	0.12±0.01	–0.8±0.2	7.6	Colloid + precipitate
PBS buffer	–	2400±300	0.14±0.03	–24±1	7.4	Precipitate
1M NaCl	Albumin	5400±200	0.17±0.08	–10±1	6.7	Precipitate
0.5M NaCl	Albumin	5400±300	0.2±0.2	–9.2±0.8	6.6	Precipitate
0.1M NaCl	Albumin	2000±500	0.25±0.07	–7.1±0.4	6.8	Precipitate
0.01M NaCl	Albumin	410±10	0.523±0.007	–13.9±0.6	7.1	Precipitate
Water	Albumin	107.6±0.4	0.13±0.02	–7.7±0.3	7.3	Colloid + precipitate
PBS buffer	Albumin	640±10	0.20±0.02	–13.2±0.5	7.6	Precipitate

Table S5: Behavior of γ-$Fe_2O_3^{\oplus}$@P(HPMA-*co*-GLM) in various conditions.

Conditions	Albumin	D_h (nm)	*PI*	ζ (mV)	pH	Description of the behavior
1M NaCl	–	166±2	0.21±0.02	–4.5±0.6	5.6	Precipitate
0.5M NaCl	–	151±2	0.209±0.06	–4±2	5.0	Colloid + precipitate
0.1M NaCl	–	150±2	0.18±0.01	–4.6±0.3	4.8	Colloid + precipitate
0.01M NaCl	–	161.9±0.7	0.24±0.01	–7.2±0.5	4.4	Colloid + precipitate
Water	–	161±6	0.24±0.05	–11.8±0.9	4.6	Colloid + precipitate
PBS buffer	–	2600±300	0.2±0.2	–22±1	7.4	Precipitate
1M NaCl	Albumin	155±1	0.18±0.01	–5±3	5.9	Colloid + precipitate
0.5M NaCl	Albumin	144±2	0.152±0.08	–5±1	5.8	Colloid + precipitate
0.1M NaCl	Albumin	143±1	0.17±0.02	–4.9±0.4	5.2	Colloid + precipitate
0.01M NaCl	Albumin	152±3	0.23±0.03	–7.2±0.2	5.1	Colloid + precipitate
Water	Albumin	156±1	0.209±0.007	–7.5±0.3	5.1	Colloid + precipitate
PBS buffer	Albumin	1500±100	0.34±0.04	–13.8±0.2	7.4	Precipitate

Table S6: Behavior of γ-$Fe_2O_3^{\ominus}$@P(HPMA-*co*-GLM) in various conditions.

Conditions	Albumin	*D_h* (nm)	*PI*	ζ (mV)	pH	Description of the behavior
1M NaCl	–	6000±800	0.2±0.1	–11±2	6.7	Precipitate
0.5M NaCl	–	9000±3000	0.6±0.3	–8±0.2	6.4	Precipitate
0.1M NaCl	–	6000±2000	0.5±0.5	–13.9±0.8	6.7	Precipitate
0.01M NaCl	–	107.9±0.7	0.146±0.007	–19.6±0.5	6.4	Colloid
Water	–	103.1±0.8	0.166±0.006	–39.2±0.8	7.1	Colloid
PBS buffer	–	1900±300	0.19±0.07	–25±1	7.4	Precipitate
1M NaCl	Albumin	8000±2000	0.8±0.2	–9±2	7.0	Precipitate
0.5M NaCl	Albumin	5900±300	0.6±0.3	–10.7±0.5	6.8	Precipitate
0.1M NaCl	Albumin	1350±70	0.24±0.07	–10.4±0.1	6.5	Precipitate
0.01M NaCl	Albumin	114.0±0.4	0.150±0.004	–18±2	7.0	Colloid
Water	Albumin	106.3±0.3	0.136±0.002	–35.1±0.6	7.2	Colloid
PBS buffer	Albumin	470±7	0.252±0.009	–13.1±0.8	7.4	Precipitate

Table S7: Behavior of γ-$Fe_2O_3^{\oplus}$@P(HPMA-*co*-AEM) in various conditions.

Conditions	Albumin	*D_h* (nm)	*PI*	ζ (mV)	pH	Description of the behavior
1M NaCl	–	590±30	0.6±0.1	–2.8±0.9	5.8	Precipitate
0.5M NaCl	–	250±7	0.271±0.006	–3±2	5.4	Precipitate
0.1M NaCl	–	129.2±0.9	0.12±0.02	–1.19±0.09	5.5	Colloid + precipitate
0.01M NaCl	–	127.8±0.3	0.1±0.01	1.2±0.3	5.6	Colloid
Water	–	133±2	0.123±0.07	22.6±0.2	4.8	Colloid
PBS buffer	–	2700±400	0.2±0.06	–22±1	7.4	Precipitate
1M NaCl	Albumin	162±3	0.15±0.04	–5±3	5.9	Colloid + precipitate
0.5M NaCl	Albumin	129.9±0.1	0.13±0.01	–8±1	6.7	Colloid + precipitate
0.1M NaCl	Albumin	124±1	0.14±0.03	–4.7±0.5	6.0	Colloid
0.01M NaCl	Albumin	360±80	0.51±0.06	1±2	5.5	Precipitate
Water	Albumin	294±9	0.38±0.03	4.7±0.1	5.4	Precipitate
PBS buffer	Albumin	2400±100	0.26±0.01	–14±1	7.4	Precipitate

Table S8: Behavior of γ-$Fe_2O_3^{\ominus}$@P(HPMA-*co*-AEM) in various conditions.

Conditions	Albumin	*D_h* (nm)	*PI*	ζ (mV)	pH	Description of the behavior
1M NaCl	–	7000±3000	0.7±0.3	–13±0.9	7.0	Precipitate
0.5M NaCl	–	9000±4000	0.7±0.5	–14.1±0.5	6.9	Precipitate
0.1M NaCl	–	10000±2000	0.7±0.5	–14.3±0.8	6.8	Precipitate
0.01M NaCl	–	138±2	0.16±0.02	–18.9±0.9	6.9	Colloid + precipitate
Water	–	94.7±0.4	0.158±0.008	–33.1±0.9	7.8	Colloid + precipitate
PBS buffer	–	1700±200	0.35±0.04	–25±1	7.4	Precipitate
1M NaCl	Albumin	5000±1000	0.5±0.5	–10.4±0.5	6.9	Precipitate
0.5M NaCl	Albumin	4200±900	0.3±0.2	–12±2	6.9	Precipitate
0.1M NaCl	Albumin	1250±30	0.28±0.04	–15.6±0.4	6.9	Precipitate
0.01M NaCl	Albumin	102.9±0.4	0.13±0.02	–23.0±0.9	7.1	Colloid
Water	Albumin	98.3±0.9	0.15±0.02	–32±2	7.8	Colloid
PBS buffer	Albumin	620±10	0.18±0.03	–13.3±0.9	7.4	Precipitate

Table S9: Behavior of γ-$Fe_2O_3^{\oplus}$@P(HPMA-*co*-HAM) in various conditions.

Conditions	Albumin	D_h (nm)	*PI*	ζ (mV)	pH	Description of the behavior
1M NaCl	–	173±2	0.161±0.005	–2±1	5.2	Colloid + precipitate
0.5M NaCl	–	144±0.4	0.13±0.01	–3±1	5.5	Colloid + precipitate
0.1M NaCl	–	125±2	0.15±0.01	0.5±0.2	4.4	Colloid
0.01M NaCl	–	122.3±0.7	0.10±0.02	5.1±0.2	4.6	Colloid
Water	–	130±3	0.130±0.006	21.9±0.7	4.6	Colloid
PBS buffer	–	3800±400	0.2±0.1	–21±2	7.4	Precipitate
1M NaCl	Albumin	139.5±0.4	0.13±0.01	–4.2±0.3	5.6	Colloid
0.5M NaCl	Albumin	124±1	0.12±0.02	–4±1	6.4	Colloid
0.1M NaCl	Albumin	120.2±0.9	0.117±0.009	–2.5±0.4	5.5	Colloid
0.01M NaCl	Albumin	129±0.7	0.12±0.01	0.6±0.6	5.8	Colloid + precipitate
Water	Albumin	123±2	0.11±0.03	9.8±0.5	5.2	Colloid
PBS buffer	Albumin	1500±100	0.34±0.01	–13.3±0.6	7.4	Precipitate

Table S10: Behavior of γ-$Fe_2O_3^{\ominus}$@P(HPMA-*co*-HAM) in various conditions.

Conditions	Albumin	D_h (nm)	*PI*	ζ (mV)	pH	Description of the behavior
1M NaCl	–	4400±700	0.7±0.5	–5.3±0.3	6.9	Precipitate
0.5M NaCl	–	8000±3000	0.8±0.3	–4±1	6.8	Precipitate
0.1M NaCl	–	161±2	0.16±0.03	–3.79±0.04	6.9	Colloid + precipitate
0.01M NaCl	–	106.0±0.6	0.120±0.004	–7.0±0.1	9.7	Colloid + precipitate
Water	–	111.6±0.2	0.13±0.01	–26±1	7.7	Colloid
PBS buffer	–	2100±200	0.23±0.04	–18±1	7.4	Precipitate
1M NaCl	Albumin	241±2	0.25±0.02	–9±1	6.8	Precipitate
0.5M NaCl	Albumin	130±2	0.120±0.009	–3±2	6.6	Colloid
0.1M NaCl	Albumin	122.0±0.5	0.13±0.02	–11.4±0.7	6.9	Colloid
0.01M NaCl	Albumin	100.8±0.6	0.13±0.01	–16±2	6.8	Colloid
Water	Albumin	114.5±0.4	0.12±0.02	–23.8±0.6	7.6	Colloid
PBS buffer	Albumin	528±9	0.24±0.01	–12±1	7.4	Precipitate

Table S11: Behavior of γ-$Fe_2O_3^{\oplus}$@P(HPMA-*co*-HAO) in various conditions.

Conditions	Albumin	D_h (nm)	*PI*	ζ (mV)	pH	Description of the behavior
1M NaCl	–	120.3±0.5	0.128±0.005	–2±1	5.1	Colloid
0.5M NaCl	–	115±1	0.11±0.01	–3±1	5.4	Colloid
0.1M NaCl	–	114.6±0.9	0.12±0.02	0.4±0.2	4.5	Colloid
0.01M NaCl	–	116±1	0.13±0.01	5.6±0.4	4.6	Colloid
Water	–	118±2	0.141±0.009	23.2±0.2	4.7	Colloid
PBS buffer	–	114.1±0.2	0.13±0.08	–5±0.5	7.4	Colloid
1M NaCl	Albumin	123±2	0.130±0.006	–4±1	5.6	Colloid
0.5M NaCl	Albumin	117.0±0.2	0.107±0.005	–2.8±0.7	5.9	Colloid
0.1M NaCl	Albumin	119±2	0.14±0.01	–1.8±0.2	5.6	Colloid
0.01M NaCl	Albumin	120.8±0.4	0.10±0.02	2±1	5.7	Colloid
Water	Albumin	125±1	0.13±0.02	10.9±0.1	5.8	Colloid
PBS buffer	Albumin	114.6±0.8	0.11±0.03	–6.1±0.4	7.4	Colloid + precipitate

Table S12: Behavior of γ-$Fe_2O_3^{\ominus}$@P(HPMA-*co*-HAO) in various conditions.

**Conditions**	**Albumin**	**D_h (nm)**	***PI***	**ζ (mV)**	**pH**	**Description of the behavior**
1M NaCl	–	112±1	0.131±0.009	–3±1	6.8	Colloid
0.5M NaCl	–	110.6±0.8	0.15±0.01	–2±3	6.8	Colloid
0.1M NaCl	–	108.4±0.6	0.13±0.01	–3±1	6.8	Colloid
0.01M NaCl	–	104.6±0.2	0.12±0.01	–8.2±0.4	7.1	Colloid
Water	–	109.1±0.01	0.12±0.01	–28.5±0.5	7.6	Colloid
PBS buffer	–	109.3±0.6	0.115±0.008	–4.2±0.9	7.4	Colloid
1M NaCl	Albumin	110.3±0.8	0.12±0.01	–5.0±0.4	6.8	Colloid
0.5M NaCl	Albumin	111±1	0.14±0.01	–4±2	6.8	Colloid
0.1M NaCl	Albumin	107±1	0.12±0.01	–3.5±0.2	6.5	Colloid
0.01M NaCl	Albumin	104±1	0.13±0.01	–7±1	6.7	Colloid
Water	Albumin	111.3±0.6	0.12±0.02	–26.6±0.5	7.6	Colloid
PBS buffer	Albumin	108±0.03	0.127±0.004	–5±1	7.4	Colloid + precipitate

Table S13: Behavior study of selected particles according to pH changes.

**Nanoparticles**	**Conditions**	**D_h (nm)**	***PI***	**ζ (mV)**	**Description of behavior**
γ-$Fe_2O_3^{\oplus}$	pH 4	10000±4000	0.6±0.4	–9.6±0.3	Precipitate
γ-$Fe_2O_3^{\oplus}$	pH 5	8000±3000	0.2±0.1	–17±1	Precipitate
γ-$Fe_2O_3^{\oplus}$	pH 6	6000±1000	0.4±0.2	–22.2±0.3	Precipitate
γ-$Fe_2O_3^{\oplus}$	pH 7	4400±700	0.3±0.2	–23.9±0.7	Precipitate
γ-$Fe_2O_3^{\oplus}$	pH 8	5000±1000	0.3±0.2	–24±1	Precipitate
γ-$Fe_2O_3^{\oplus}$	pH 9	5000±1000	0.34±0.01	–23.5±0.4	Precipitate
γ-$Fe_2O_3^{\oplus}$	pH 10	4400±200	0.3±0.3	–23±1	Precipitate
γ-$Fe_2O_3^{\oplus}$@P(HPMA)	pH 4	5000±500	0.24±0.06	–8.8±0.7	Precipitate
γ-$Fe_2O_3^{\oplus}$@P(HPMA)	pH 5	6200±600	0.4±0.2	–14.9±0.7	Precipitate
γ-$Fe_2O_3^{\oplus}$@P(HPMA)	pH 6	6300±500	0.7±0.5	–21.3±0.6	Precipitate
γ-$Fe_2O_3^{\oplus}$@P(HPMA)	pH 7	6000±1000	0.5±0.5	–22±1	Precipitate
γ-$Fe_2O_3^{\oplus}$@P(HPMA)	pH 8	3500±300	0.2±0.1	–21.7±0.9	Precipitate
γ-$Fe_2O_3^{\oplus}$@P(HPMA)	pH 9	3300±500	0.2±0.1	–21.7±0.8	Precipitate
γ-$Fe_2O_3^{\oplus}$@P(HPMA)	pH 10	4000±1000	0.3±0.2	–22±1	Precipitate
γ-$Fe_2O_3^{\oplus}$@P(HPMA-*co*-HAO)	pH 4	111.9±0.3	0.133±0.009	–3±1	colloid
γ-$Fe_2O_3^{\oplus}$@P(HPMA-*co*-HAO)	pH 5	109.9±0.1	0.11±0.01	–3.2±0.7	colloid
γ-$Fe_2O_3^{\oplus}$@P(HPMA-*co*-HAO)	pH 6	114±2	0.10±0.02	–4±1	colloid
γ-$Fe_2O_3^{\oplus}$@P(HPMA-*co*-HAO)	pH 7	112±0.1	0.118±0.009	–4±1	colloid
γ-$Fe_2O_3^{\oplus}$@P(HPMA-*co*-HAO)	pH 8	115±0.8	0.14±0.02	–5±1	colloid
γ-$Fe_2O_3^{\oplus}$@P(HPMA-*co*-HAO)	pH 9	111±0.8	0.1±0.02	–6±2	colloid
γ-$Fe_2O_3^{\oplus}$@P(HPMA-*co*-HAO)	pH 10	117±1	0.13±0.01	–7.3±0.9	colloid

Table S14: Behavior study of selected particles according to pH changes.

**Nanoparticles**	**Conditions**	***D_h* (nm)**	***PI***	***ζ* (mV)**	**Description of behavior**
γ-$Fe_2O_3^{⊖}$	pH 4	4000±500	0.2±0.2	–17±1	Precipitate
γ-$Fe_2O_3^{⊖}$	pH 5	2800±300	0.3±0.1	–21±1	Precipitate
γ-$Fe_2O_3^{⊖}$	pH 6	2220±200	0.4±0.1	–24±1	Precipitate
γ-$Fe_2O_3^{⊖}$	pH 7	1900±200	0.38±0.08	–25±2	Precipitate
γ-$Fe_2O_3^{⊖}$	pH 8	2700±200	0.4±0.2	–24±1	Precipitate
γ-$Fe_2O_3^{⊖}$	pH 9	2600±100	0.4±0.1	–23.3±0.4	Precipitate
γ-$Fe_2O_3^{⊖}$	pH 10	4600±300	0.13±0.09	–23±1	Precipitate
γ-$Fe_2O_3^{⊖}$@P(HPMA)	pH 5	2200±200	0.45±0.08	–18±2	Precipitate
γ-$Fe_2O_3^{⊖}$@P(HPMA)	pH 6	1800±200	0.32±0.01	–25.3±0.9	Precipitate
γ-$Fe_2O_3^{⊖}$@P(HPMA)	pH 7	2400±300	0.4±0.1	–25±2	Precipitate
γ-$Fe_2O_3^{⊖}$@P(HPMA)	pH 8	2000±300	0.4±0.2	–24.7±0.8	Precipitate
γ-$Fe_2O_3^{⊖}$@P(HPMA)	pH 9	2900±300	0.4±0.2	–24±2	Precipitate
γ-$Fe_2O_3^{⊖}$@P(HPMA)	pH 10	2300±200	0.29±0.01	–23±2	Precipitate
γ-$Fe_2O_3^{⊖}$@P(HPMA-*co*-HAO)	pH 4	102.2±0.2	0.148±0.005	–4±1	colloid
γ-$Fe_2O_3^{⊖}$@P(HPMA-*co*-HAO)	pH 5	102.4±0.5	0.131±0.005	–5±2	colloid
γ-$Fe_2O_3^{⊖}$@P(HPMA-*co*-HAO)	pH 6	102.9±0.5	0.124±0.002	–4±1	colloid
γ-$Fe_2O_3^{⊖}$@P(HPMA-*co*-HAO)	pH 7	104.4±0.6	0.125±0.009	–3.6±0.8	colloid
γ-$Fe_2O_3^{⊖}$@P(HPMA-*co*-HAO)	pH 8	104.3±0.7	0.131±0.007	–5±1	colloid
γ-$Fe_2O_3^{⊖}$@P(HPMA-*co*-HAO)	pH 9	104.3±0.8	0.116±0.009	–5±1	colloid
γ-$Fe_2O_3^{⊖}$@P(HPMA-*co*-HAO)	pH 10	103±1	0.13±0.01	–6.6±0.3	colloid

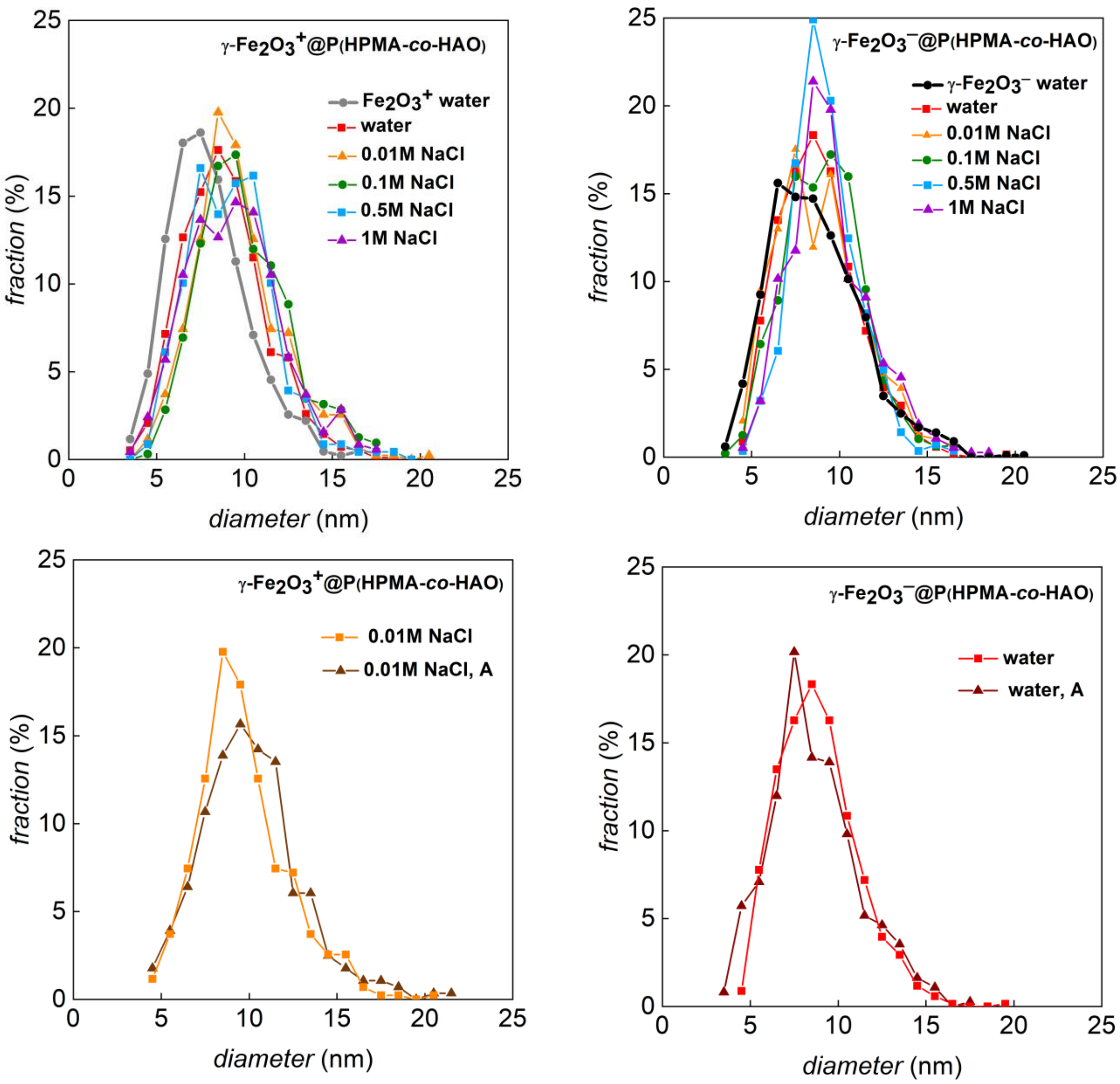

Scheme S1: Diameter changes of the magnetic particles coated by P(HPMA-*co*-HAO) depending on different conditions. "A" refers to presence of Albumin.

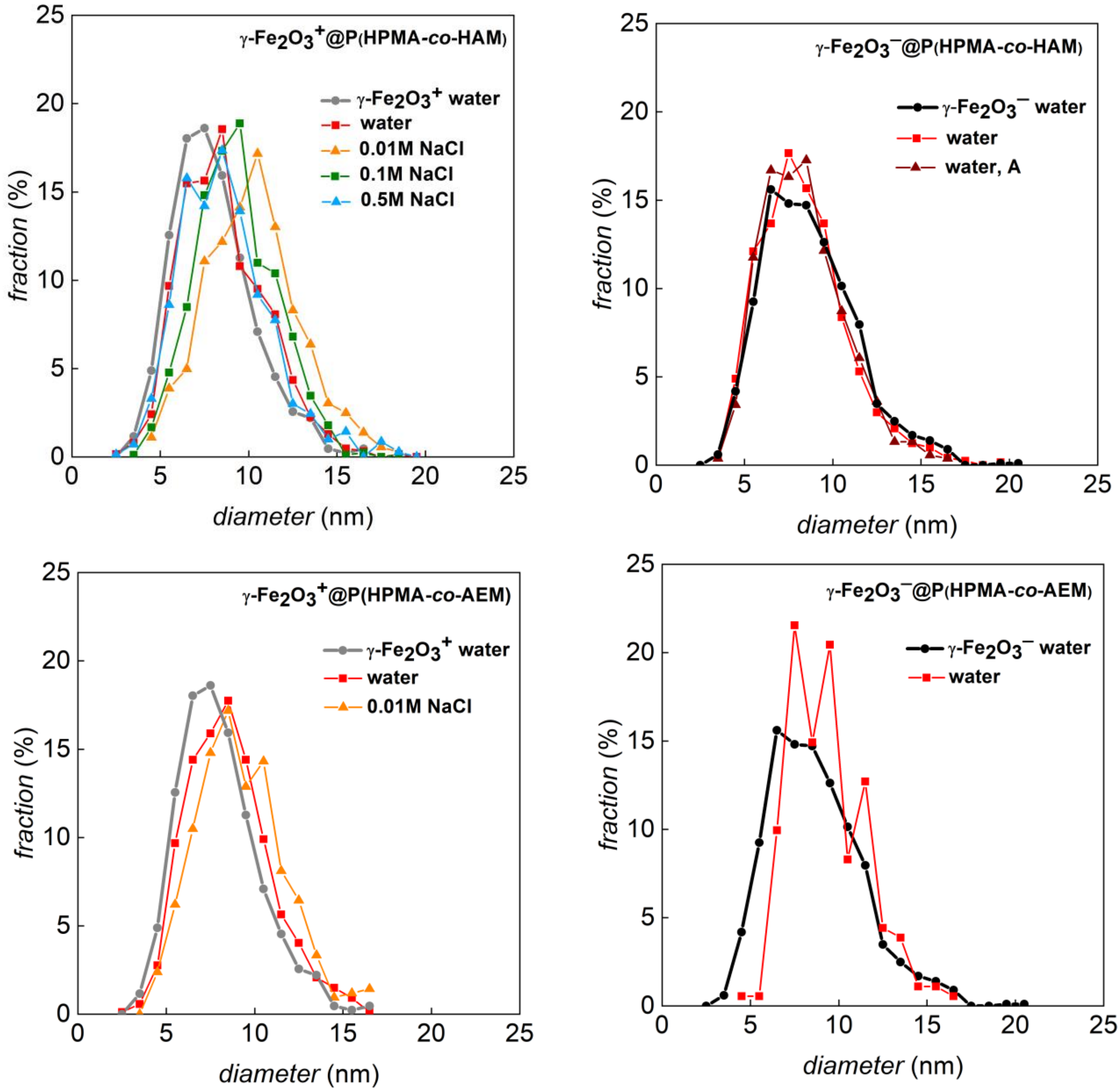

Scheme S2: Diameter changes of the magnetic particles coated by P(HPMA-*co*-HAM) and P(HPMA-*co*-AEM) depending on different conditions. "A" refers to presence of Albumin.

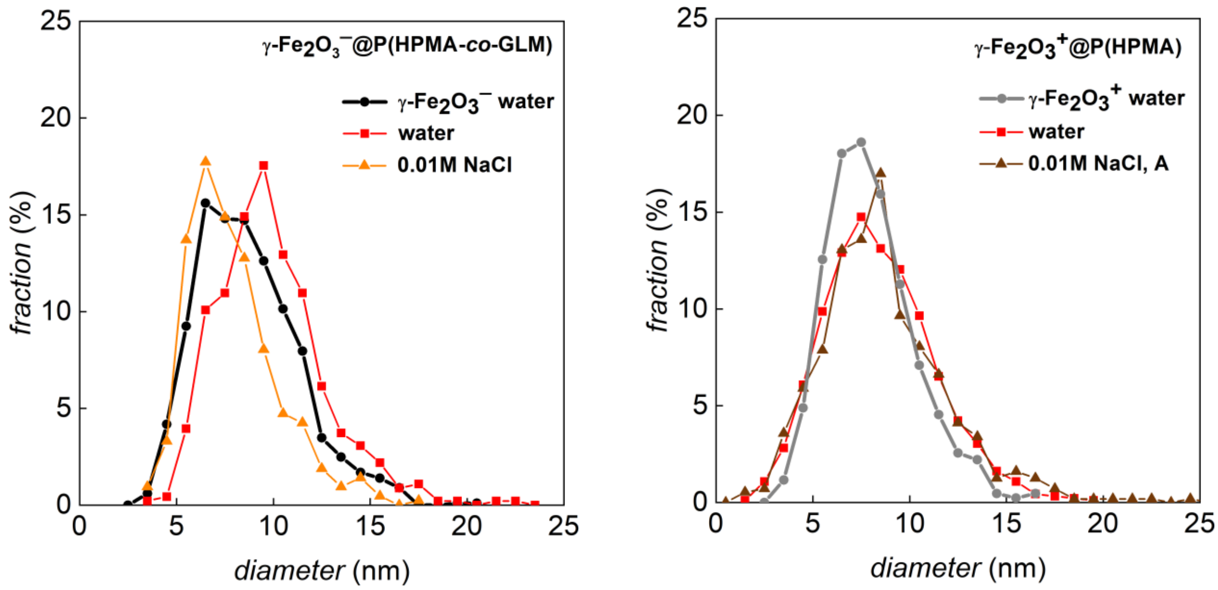

Scheme S3: Diameter changes of the magnetic particles coated by P(HPMA-*co*-GLM) and P(HPMA) depending on different conditions. "A" refers to presence of Albumin.

Table S15: Relative MR signal changes in the kidney cortex.

Nanoparticles	Time after application (Hrs)						
	**0**	**0.5**	**4**	**24**	**72**	**168**	**336**
γ-$Fe_2O_3^{\oplus}$	1.0 ± 0.2	1.04 ± 0.06	1.09 ± 0.07	1.05 ± 0.08	1.03 ± 0.03	1.11 ± 0.06	1.04 ± 0.05
γ-$Fe_2O_3^{\ominus}$	1.000 ± 0.004	0.97 ± 0.03	0.99 ± 0.01	1.13 ± 0.07	0.98 ± 0.01	1.056 ± 0.02	0.96 ± 0.02
γ-$Fe_2O_3^{\oplus}$@P(HPMA-*co*-HAO)	1.00 ± 0.05	0.76 ± 0.05	1.00 ± 0.06	0.98 ± 0.01	0.90 ± 0.02	1.02 ± 0.03	0.92 ± 0.07
γ-$Fe_2O_3^{\ominus}$@P(HPMA-*co*-HAO)	1.00 ± 0.02	0.85 ± 0.05	1.00 ± 0.03	1.08 ± 0.05	1.06 ± 0.03	1.06 ± 0.04	1.03 ± 0.02
Resovist®	1.00 ± 0.01	0.81 ± 0.08	1.00 ± 0.02	1.053 ± 0.005	1.02 ± 0.02	1.017 ± 0.005	1.07 ± 0.09

Table S16: Relative MR signal changes in the kidney medulla.

Nanoparticles	Time after application (Hrs)						
	**0**	**0.5**	**4**	**24**	**72**	**168**	**336**
γ-$Fe_2O_3^{\oplus}$	1.0 ± 0.2	1.08 ± 0.05	1.20 ± 0.06	1.16 ± 0.07	1.07 ± 0.03	1.07 ± 0.05	1.06 ± 0.05
γ-$Fe_2O_3^{\ominus}$	1.00 ± 0.05	0.94 ± 0.03	1.03 ± 0.02	1.13 ± 0.07	0.99 ± 0.02	1.04 ± 0.03	0.99 ± 0.04
γ-$Fe_2O_3^{\oplus}$@P(HPMA-*co*-HAO)	1.00 ± 0.07	0.81 ± 0.03	1.1 ± 0.1	0.97 ± 0.03	0.92 ± 0.01	1.05 ± 0.06	0.99 ± 0.07
γ-$Fe_2O_3^{\ominus}$@P(HPMA-*co*-HAO)	1.00 ± 0.02	0.91 ± 0.05	0.98 ± 0.03	1.09 ± 0.06	1.04 ± 0.02	1.06 ± 0.04	1.08 ± 0.02
Resovist®	1.00 ± 0.02	1.1 ± 0.1	1.08 ± 0.04	1.13 ± 0.03	1.16 ± 0.03	1.21 ± 0.03	1.1 ± 0.1

Table S17: Relative MR signal changes in the liver.

Nanoparticles	Time after application (Hrs)						
	**0**	**0.5**	**4**	**24**	**72**	**168**	**336**
γ-$Fe_2O_3^{\oplus}$	1.0 ± 0.2	0.15 ± 0.05	0.18 ± 0.06	0.16 ± 0.07	0.15 ± 0.03	0.20 ± 0.05	0.48 ± 0.05
γ-$Fe_2O_3^{\ominus}$	1.00 ± 0.05	0.16 ± 0.03	0.16 ± 0.02	0.18 ± 0.07	0.16 ± 0.02	0.25 ± 0.03	0.36 ± 0.04
γ-$Fe_2O_3^{\oplus}$@P(HPMA-*co*-HAO)	1.00 ± 0.07	0.34 ± 0.03	0.3 ± 0.1	0.28 ± 0.03	0.31 ± 0.01	0.47 ± 0.07	0.63 ± 0.07
γ-$Fe_2O_3^{\ominus}$@P(HPMA-*co*-HAO)	1.00 ± 0.02	0.15 ± 0.05	0.15 ± 0.05	0.17 ± 0.06	0.15 ± 0.02	0.25 ± 0.04	0.41 ± 0.02
Resovist®	1.00 ± 0.02	0.2 ± 0.1	0.17 ± 0.04	0.17 ± 0.03	0.19 ± 0.03	0.19 ± 0.03	0.2 ± 0.1

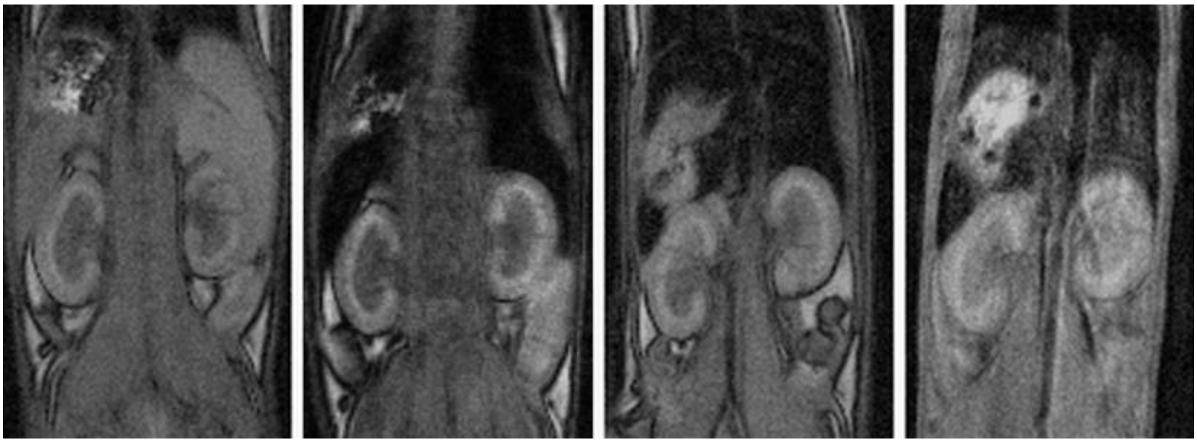

(a) (b) (c) (d)

Figure S1: *In vivo* MRI scans (gradient echo sequence with a mixed T1/T2* contrast) of the kidney before nanoparticle (γ-Fe_2O_3⊕) application (**a**), immediately after (**b**), one day after (**c**), and 2 weeks after application (**d**).
